# Microneedle–nanoparticle hybrid platforms for metabolic syndrome: advances in point-of-care diagnostics and transdermal therapeutics

**DOI:** 10.1186/s11671-025-04376-7

**Published:** 2025-10-28

**Authors:** Jaewon Choi, Sanghyeok Jang, Seona Yu, Yu-Rim Ahn, Minse Kim, Hyungseok Lee, Hyun-Ouk Kim

**Affiliations:** 1 Division of Chemical Engineering and Bioengineering, College of Art, Culture and Engineering, Kangwon National University, Gangwon State, Chuncheon, 24341 Republic of Korea; 2 Department of Smart Health Science and Technology, College of Engineering, Kangwon National University, Gangwon State, Chuncheon, 24341 Republic of Korea; 3 Department of Mechanical and Biomedical Engineering, College of Engineering, Kangwon National University, Gangwon State, Chuncheon, 24341 Republic of Korea; 4 Institute of Fermentation of Brewing, Kangwon National University, Chuncheon, 24341 Republic of Korea

**Keywords:** Microneedle-based delivery, Nanoparticle integration, Point-of-care diagnostics, Transdermal drug administration, Metabolic syndrome therapy, Minimally invasive systems, Hybrid nanostructures

## Abstract

Metabolic syndrome encompasses various conditions, including diabetes, obesity, and metabolic diseases. It is characterized by high blood sugar, abnormal cholesterol, and high blood pressure levels. Research has investigated combined methods using microneedles and nanoparticles for diagnosis and treatment to improve effectiveness while reducing harm to healthy tissues. This process allows potential improvements in diagnostic capabilities for detecting biomarkers associated with metabolic diseases. Using microneedles in conjunction with nanoparticles offers a promising avenue for advancements in metabolic disease management. With the potential for targeted and precise delivery of therapeutic agents, this integrated approach could revolutionize treatment strategies. Combining these technologies not only enhances the delivery and efficacy of existing treatments but also paves the way for new therapeutic modalities. The careful management and focused release of therapeutic agents made possible by this method could help create new treatment options that weren't possible before. As we learn more about this field, exploring the potential challenges and limitations associated with integrating microneedles and nanoparticles is crucial for metabolic diseases. Understanding these aspects is fundamental to guiding further advancement and ensuring the development of safe, effective, and patient-centric interventions.

**Supplementary Information:**

The online version contains supplementary material available at 10.1186/s11671-025-04376-7.

## Introduction

**Fig. 1:**
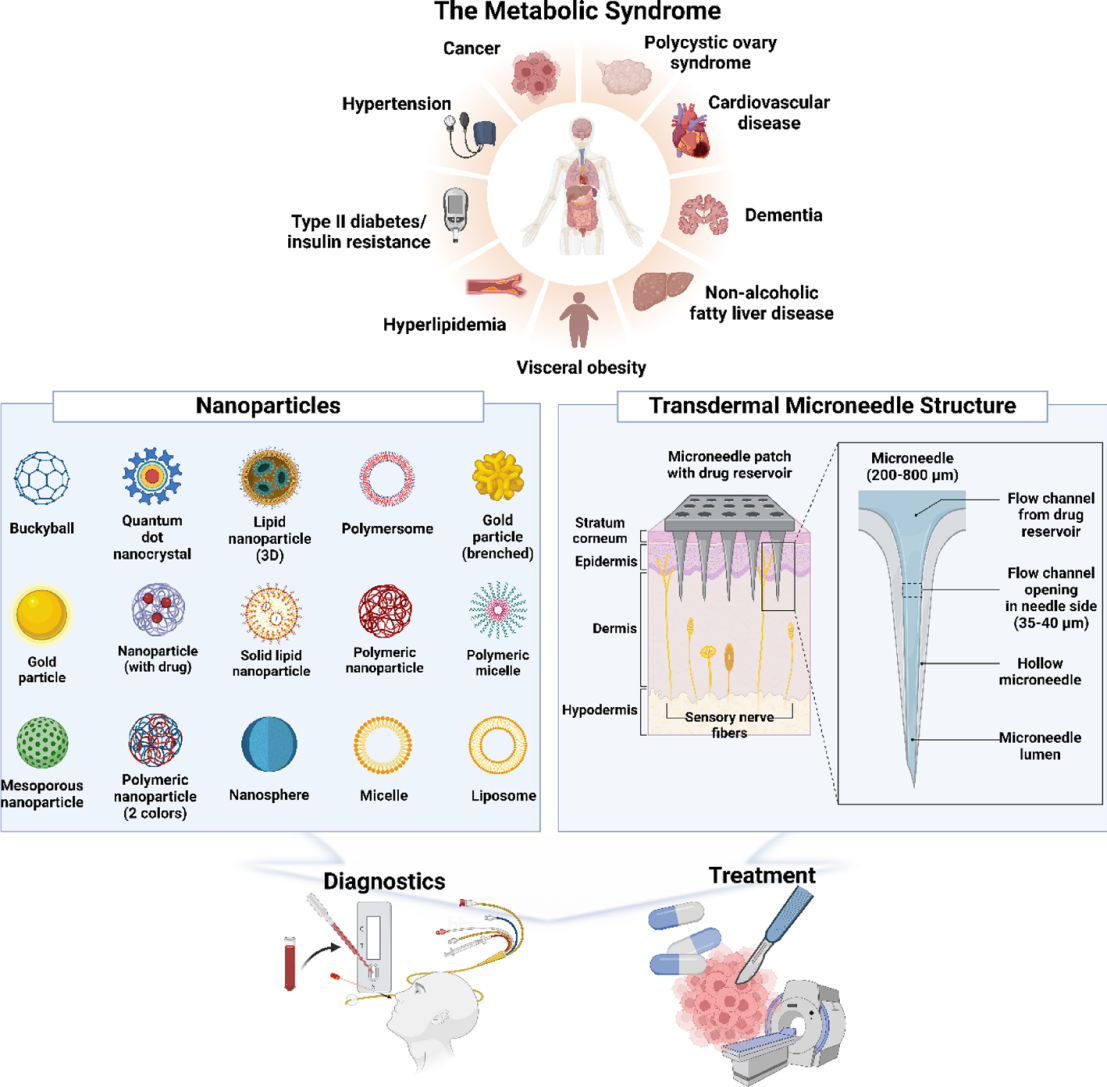
A schematic diagram illustrates the diagnosis and treatment of metabolic syndromes using a platform that fused nanoparticles and microneedles. Encapsulating various organic and inorganic nanoparticles in microneedles enables its application. Created with BioRender.com

Obesity, diabetes, hypertension, dyslipidemia, and other interconnected conditions that disturb metabolic balance and markedly increase the risk of heart disease, stroke, and other chronic consequences are together referred to as metabolic syndrome [1–4]. Due to sedentary lifestyles, dietary imbalances, and aging populations, the prevalence of these disorders has significantly increased worldwide [5–7]. They are characterized by raised blood glucose, aberrant lipid profiles, and increased blood pressure [8–10]. In order to manage metabolic diseases and avoid permanent organ damage and long-term repercussions, early diagnosis and prompt intervention are essential. However, centralized laboratory testing, venous blood collection, and intermittent monitoring are frequently used in conventional diagnostic techniques, which may lead to poor patient compliance and delayed diagnosis [11–13]. From oral drugs and insulin injections to surgery, the majority of today's therapies are linked to systemic adverse effects, high expenses, and limited accessibility for patients in low-resource environments [14–17]. Recent studies have concentrated on integrating microneedles (MNs) and nanoparticles (NPs) into multipurpose platforms that allow for precise, patient-friendly, and minimally invasive disease management in order to overcome these obstacles [18–20]. Because microneedles can enter the stratum corneum painlessly and reach interstitial fluid (ISF), they offer a means of delivering therapeutics and collecting biomarkers [21–23]. These technologies provide notable benefits in terms of real-time monitoring, targeted release, and multiplexed sensing capabilities when paired with functionalized nanoparticles, which are able to sense, respond, and deliver payloads at the molecular level [24–26]. By allowing for stimuli-responsive behaviors, programmable drug release profiles, and flexibility to specific pathophysiological situations, MN–NP systems in particular have shown promise for customized therapy [27–29]. In addition to improving therapeutic results, this combination of nanotechnology and transdermal distribution makes it easier to identify biomarkers in ISF, which leads to an earlier and more precise diagnosis [30, 31]. Despite these benefits, several challenges still exist. Clinical translation requires consideration of long-term biocompatibility, manufacturing scalability, integration with digital health technologies, and regulatory standardization [32–34]. Furthermore, systems that can adjust to changing biomarker levels, multi-target circumstances, and feedback-controlled therapies are necessary due to the dynamic nature of metabolic disorders [35, 36]. In order to diagnose and treat metabolic syndrome, this review attempts to give a thorough summary of recent developments in microneedle–nanoparticle integrated systems. This review looks at early research and real-life examples, specific designs for different uses, the basic ideas behind microneedles and nanoparticles, and the future of easy-to-use technologies for treating metabolic diseases. Figure 1 conceptually illustrates an overview of the diagnostic and therapeutic applications of the MN–NP platform, providing a visual context for the subsequent sections.

## Types of microneedles

**Fig. 2:**
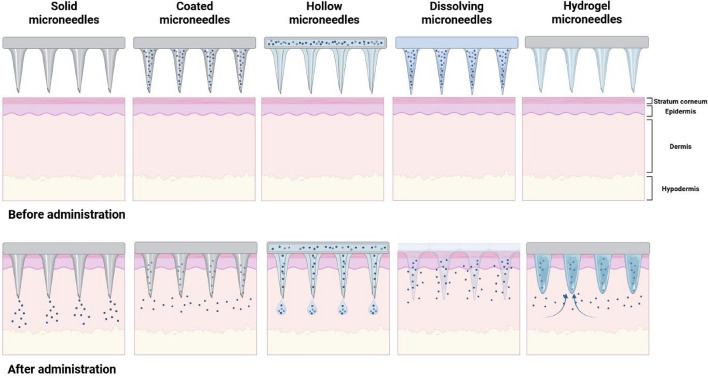
Schematic of different types of microneedles and their features. The structures of solid, coated, hollow, and dissolving microneedles differ. Each microneedle type has different drug delivery properties. Solid microneedles are suitable for increased penetration and drug permeability. The hollow microneedle forms a path for drug injection. The coated microneedle is characterized by containing the drug. Created with BioRender.com

Microneedle technology includes various types of microneedles that can be combined with nanoparticles to prevent, diagnose, and treat metabolic diseases, such as diabetes. These include solid, coated, dissolving, hollow, and hydrogel-forming microneedles (Fig. 2) [37–39]. This section briefly describes the types and characteristics of microneedles.

### Solid microneedles

Solid microneedles are entirely composed of a biocompatible material such as silicon or metal and are designed to physically puncture the skin barrier, allowing enhanced drug delivery into the systemic circulation [40]. Advances in technology have enabled the fabrication of solid microneedles from various materials such as silicone, stainless steel, glass, and iron. The shape, depth, and width of the microneedle determine the delivery rate [41–43]. Solid microneedles can deliver various therapeutics and nanoparticles transdermally; however, many studies have shown that nanoparticles are retained in the stratum corneum [44, 45]. A. Ovsianikov et al. reported the production of Ormocer® microneedles using a two-photon polymerization technique at various aspect ratios. These microneedles could penetrate the porcine adipose tissue without cracking, which may pose a risk of skin damage and infection, owing to their piercing nature [46]. Using metal or silicon in these microneedles could also raise concerns regarding potential allergic reactions in some individuals [47]. Alternative fabrication techniques like electro-drawing (ED) have arisen to address these challenges. ED makes biodegradable polymer microneedles from liquid polymer at room temperature by using electric forces, which means it doesn't need molds or high heat. This method encapsulates various therapeutic agents, streamlines production, and ensures precise geometric control [48]. We could further investigate the long-term effects and safety of using solid microneedles for drug delivery into the systemic circulation. Solid microneedles demonstrate impressive mechanical strength and precision, but we still require more information about their long-term compatibility in ongoing applications. Standardized mechanical testing is necessary to ensure homogeneous microneedle geometry, which directly influences drug transport efficiency and penetration depth. Future research could incorporate biosensors into the tips of needles, creating various devices capable of real-time monitoring and delivery.

### Coated microneedles

Coated microneedles generally have coatings of drug solutions, polymers, small molecules, or nanoparticles [49–51]. When microneedles penetrate the skin, the drug-coated nanoparticles are released, facilitating controlled and localized drug delivery. Coated microneedles use techniques such as dip coating, gas jet drying, spray coating, and piezoelectric inkjet printing to deposit drug-containing nanoparticles on the surface of solid microneedles, increasing their stability and treatment success rate [52–54]. For example, DeMuth et al. designed poly(lactic-co-glycolic acid) microneedles coated with cationic poly(β-amino ester) and negatively charged interlayer cross-linked multilayer lipid vesicles to deliver protein antigens and antigen adjuvants [55]. Some argue that using microneedles coated with drug-containing nanoparticles may cause skin irritation and adverse reactions because of the introduction of foreign particles into the body. The long-term effects of drug-coated nanoparticles on human health and the environment are also major concerns. Recent studies show that coating thickness and uniformity greatly affect drug release rates and microneedle penetration efficiency. Fast coating techniques can speed up manufacturing but may lead to uneven drug loading and increased local immune responses with repeated use. Using biodegradable polymers or special additives in coatings can help release drugs slowly and in specific areas, reducing toxicity issues. Finally, having consistent quality control measures, particularly for how well the coating sticks and how evenly the nanoparticles are spread, is crucial for broader use in healthcare.

### Hollow microneedles

In contrast to solid microneedles, hollow microneedles feature a channel that enables the direct administration of therapeutics or nanoparticles into the dermal layers or systemic circulation [56]. This design provides accurate dosage control and can be beneficial for administering specific formulations [57–59]. For example, Mir et al. reported a liquid injection system (AdminPen®) that combined bacterial enzyme-reactive nanoparticles with hollow microneedles. In vivo skin insertion and skin motility studies showed that the system delivered approximately 8.5-fold higher concentrations of the drug carvacrol in nanoparticle form compared to a topically applied hydrogel containing pure carvacrol, indicating its potential for increasing the transdermal delivery of nanoparticles using hollow microneedles[60]. Although hollow microneedles allow precise and increased nanoparticle delivery through the skin, their complex composition limits this strategy. Hollow microneedles need precise fabrication tolerances to ensure channel integrity; even small defects can affect dose accuracy and patient safety. The rise in material costs and the risk of needle clogging during extended use present practical issues for large-scale implementation. Some studies indicate the potential for local tissue irritation from mechanical stress at the needle-skin interface when higher flow rates are necessary. Future work may concentrate on integrating advanced microfluidic designs and biocompatible coatings to improve reliability and comfort in hollow microneedle applications.

### Dissolving microneedles

Dissolving microneedles combined with nanoparticles represent a convenient and painless solution for drug delivery, enhancing the safety and effectiveness of extended treatments [61, 62]. Dissolving microneedles use materials that can penetrate and rapidly dissolve within the skin layer, specifically biocompatible, water-soluble polymers such as hyaluronic acid, polyvinylpyrrolidone, and gelatin methacryloyl [63]. This encapsulates the drug inside the matrix, which stabilizes it and facilitates long-term storage. In a recent study, the psoriasis drug methotrexate sodium salt (MTX Na), which doesn't dissolve well in water, was turned into tiny crystals and added to soluble microneedles. After being inserted into the skin, the microneedles with MTX nanocrystals showed effective drug delivery, with about 322 times more of the drug staying in the skin compared to regular MTX 24 h later. In an in vivo study in rats, approximately 12.5% of the MTX nanocrystals accumulated in the skin even 72 h after administration, indicating localized and sustained drug delivery, facilitated by the strategy of soluble microneedles and nanotechnology [64]. Dissolving microneedles offer patient comfort and targeted drug delivery, but their mechanical strength might not be adequate for deeper skin penetration in certain formulations. Researchers are examining the effects of polymer concentration and needle geometry on the dissolution rate to enhance structural integrity and controlled release. Thorough toxicological assessments are essential to prevent the accumulation of polymer residues or byproducts in vulnerable tissues. By incorporating real-time monitoring components, such as embedded sensors, their applications could be expanded beyond drug delivery to include diagnostics.

### Stimuli-responsive microneedles

Stimuli-responsive microneedles are advanced transdermal drug delivery systems engineered to release pharmaceuticals in reaction to external stimuli, such as thermal, photonic, magnetic, or electrical signals, as well as specific internal biological indicators, including fluctuations in glucose levels, pH levels, and enzymatic activity. Recent research has investigated the efficacy of glucose-responsive microneedles. Studies have shown effective regulation of blood glucose levels through automated insulin release in response to elevated glucose concentrations. Questions regarding biocompatibility, long-term stability, and reproducibility in clinical settings persist [65]. Ghavami Nejad et al. [66] described a microneedle patch composed of catechol that effectively mitigates hypoglycemic episodes by administering zinc-glucagon for a duration of up to six hours. Polydopamine-based photothermal microneedle patches have demonstrated efficacy in treating obesity by converting subcutaneous white adipose tissue into brown adipose tissue. In controlled trials, this resulted in a 19% reduction in body weight in obese animal models [67]. Despite the promising developments, thorough studies reveal persistent issues, including clinical scalability, the durability of included nanoparticles, and precise responsiveness. This indicates that more comprehensive preclinical and clinical investigations are necessary to ensure the safe application of these microneedle platforms in clinical practice [68].

Collectively, these findings affirm that microneedle–nanoparticle systems, when properly engineered, offer a well-tolerated and safe interface with biological systems. The use of immune-safe materials, targeted delivery, and temporary physical changes creates a solid basis for using these systems in medicine. Continued refinement of formulation chemistry and delivery parameters will further optimize immunological compatibility and enhance long-term applicability. To sum up the different functions and design factors of microneedle technologies, Table 1 gives a comparison of the five main types of microneedles, showing their structures, how they deliver substances, their benefits, and their drawbacks. This classification helps us understand how microneedles can be adapted and improved in hybrid systems, particularly when combined with nanoparticles for medical uses.

**Table 1:** Classification and characteristics of microneedles

Type	Structure	Mechanisms	Advantage	Limitations
Solid MN	Needle-only	Puncture skin for passive diffusion	Simple fabrication, reusable mold	No intrinsic drug loading
Coated MN	Solid + drug coating	The drug dissolves after insertion	Rapid release, surface flexibility	Low drug capacity, fragile coating
Dissolving MN	Water-soluble matrix	MN dissolves in skin	Biodegradable, no residue	Limited mechanical strength
Hollow MN	Needle + lumen	Active fluid infusion	Large-molecule delivery possible	Complex fabrication, clogging risk

### Integrated fabrication–materials–nanomaterials overview

In summary, the performance and application range of microneedles are mostly determined by the manufacturing techniques, base materials, and approaches to nanomaterial integration. Recent studies indicate that micromolding is the predominant technique, especially for the fabrication of dissolvable and hydrogel-forming microneedles made from biodegradable polymers including hyaluronic acid (HA), polyvinylpyrrolidone (PVP), and poly(lactic-co-glycolic acid) (PLGA) [69]. This approach is good for making things cheaply and in huge quantities, but it doesn't work well for making microstructures with high resolution. On the other hand, photolithography and etching-based lithographic methods have better resolution and reproducibility, which makes them good for creating solid or hollow microneedles with exact channel topologies. But these procedures are expensive and don't work as well for large-scale production [70].

Metal-based microneedles, which are usually made of stainless steel, titanium, or nickel, are very strong and can easily penetrate skin. They are commonly made using methods like laser cutting, etching, or electroplating, which make it possible to make arrays with sharp points over and over again [70]. Metal microneedles are great for applications that need to be inserted multiple times since they are stiff and can easily get through resistant skin layers. But because they don't break down and might not be biocompatible, they can't be used for lengthy periods of time. They are better for short-term distribution or diagnostic sampling [71].

Recently, three-dimensional (3D) printing has become popular for making sophisticated and unique microneedle designs, especially for systems that respond to stimuli. This method makes it easier to combine sophisticated nanomaterials including conductive polymers, stimuli-sensitive polymers, metal–organic frameworks (MOFs), and covalent organic frameworks (COFs) [72]. Thermal drawing and electro-drawing methods also make it possible to make things quickly and regulate their shape, although there are still problems with getting things to have the same size and shape [69].

The process used to make something has a direct effect on how well base materials and nanoparticles work together. For instance, dissolving microneedles constructed of biodegradable polymers are great for holding delicate biological materials like liposomes and polymeric nanoparticles [73]. On the other hand, silicon- and metal-based microneedles are more mechanically stable, which makes it easier to add stiff nanomaterials like metallic nanoparticles and MOFs [71]. Hydrogel-based microneedles can release nanomaterials in a regulated and long-lasting way because they can swell. They also work well with electrochemical nanomaterials for biosensing applications [74]. To systematically compare production methods, base materials, and nanomaterial integration across various microneedle types, we provide a summary as Table 2, which builds on the discussion in Sect. 2.

**Table 2:** Comparative overview of microneedle types, fabrication methods, base materials, nanomaterials, advantages, and limitations

Microneedle type	Fabrication methods	Base materials	Recommended nanomaterials	Advantages	Limitations
Solid	Photolithography, Etching, Laser cutting	Silicon, Metals (stainless steel, titanium, nickel)	Metallic NPs, Ceramic NPs	High mechanical strength, reliable penetration, reusable	Non-biodegradable, higher cost, limited drug loading
Coated	Dip-coating, Spray-coating, Electroplating	Metals, Silicon, Polymers	Liposomes, Polymeric NPs, Antibodies, siRNA	Precise delivery of surface-loaded therapeutics, simple design	Limited payload capacity, non-uniform coating risk
Hollow	Micromoulding, Laser drilling, Etching	Silicon, Metals, PLGA	MOFs, Polymeric NPs, Exosomes	Large-volume delivery, direct fluid injection	Risk of clogging, requires pressure control, expensive
Dissolving	Micromoulding, Casting	HA, PVP, PLGA	Liposomes, Polymeric NPs, DNA, mRNA	Biodegradable, safe, rapid drug release	Low mechanical strength, limited penetration depth
Hydrogel-forming	Photocuring, 3D printing, Mould casting	PEGDA, GelMA, PVA	Fluorescent NPs, Enzyme-loaded NPs, Metallic NPs	Swellable, sustained drug release, ISF sampling	Slow insertion due to swelling, mechanical weakness
Stimuli-responsive	3D printing, Thermal drawing, Electro-drawing	Smart polymers (PNIPAM, PDA), MOF/COF composites	Conductive polymers, MOFs, COFs	Controlled release triggered by pH, ROS, or temperature	Fabrication complexity, reproducibility challenges

To contextualize the comparative value of MN–NP platforms, Table 3 summarizes their advantages and limitations relative to conventional transdermal and diagnostic modalities.

**Table 3:** Comparative overview of MN–NP systems versus other transdermal and diagnostic approaches

Approach	Mode	What it can handle	Key advantages	Main limitations	Typical use cases
MN–NP	Transdermal delivery & ISF diagnostics	Small molecules → macromolecules (proteins, nucleic acids, vaccines); real-time sensing	Minimally invasive; tunable release via nanoparticles; localized delivery; real-time optical/electrochemical readout	Manufacturing complexity; NP/storage stability; regulatory uncertainty	Controlled delivery; continuous/multiplexed biomarker monitoring
Conventional transdermal patch	Passive diffusion across skin	Lipophilic, low-MW drugs	Simple, painless, inexpensive	Poor for hydrophilic/macromolecules; limited flux control	Chronic small-molecule dosing (e.g., nicotine, fentanyl)
Microneedles (no nanoparticles)	Transdermal delivery or sampling	Small molecules; some biotherapeutics	Minimally invasive; improved permeation vs patch	Less control over release kinetics and signal amplification	Bolus dosing; basic ISF access
Hypodermic injection	Parenteral (SC/IM/IV)	Broad (incl. biologics)	High bioavailability; established regulation	Pain; needle anxiety; systemic side effects; clinic visit	Vaccines; biologics; acute dosing
Wearable sweat/saliva sensors	Non-invasive body fluids	Electrolytes, some metabolites	Comfortable; continuous monitoring	Variable secretion rates; limited biomarker breadth; dilution effects	Fitness/physiology tracking
Laboratory blood assays	Invasive phlebotomy	Gold-standard panels	High analytical accuracy; broad panels	Requires lab; not continuous; invasive	Diagnosis, confirmation testing

## Diagnosing metabolic disorders using microneedles and nanoparticles

**Fig. 3:**
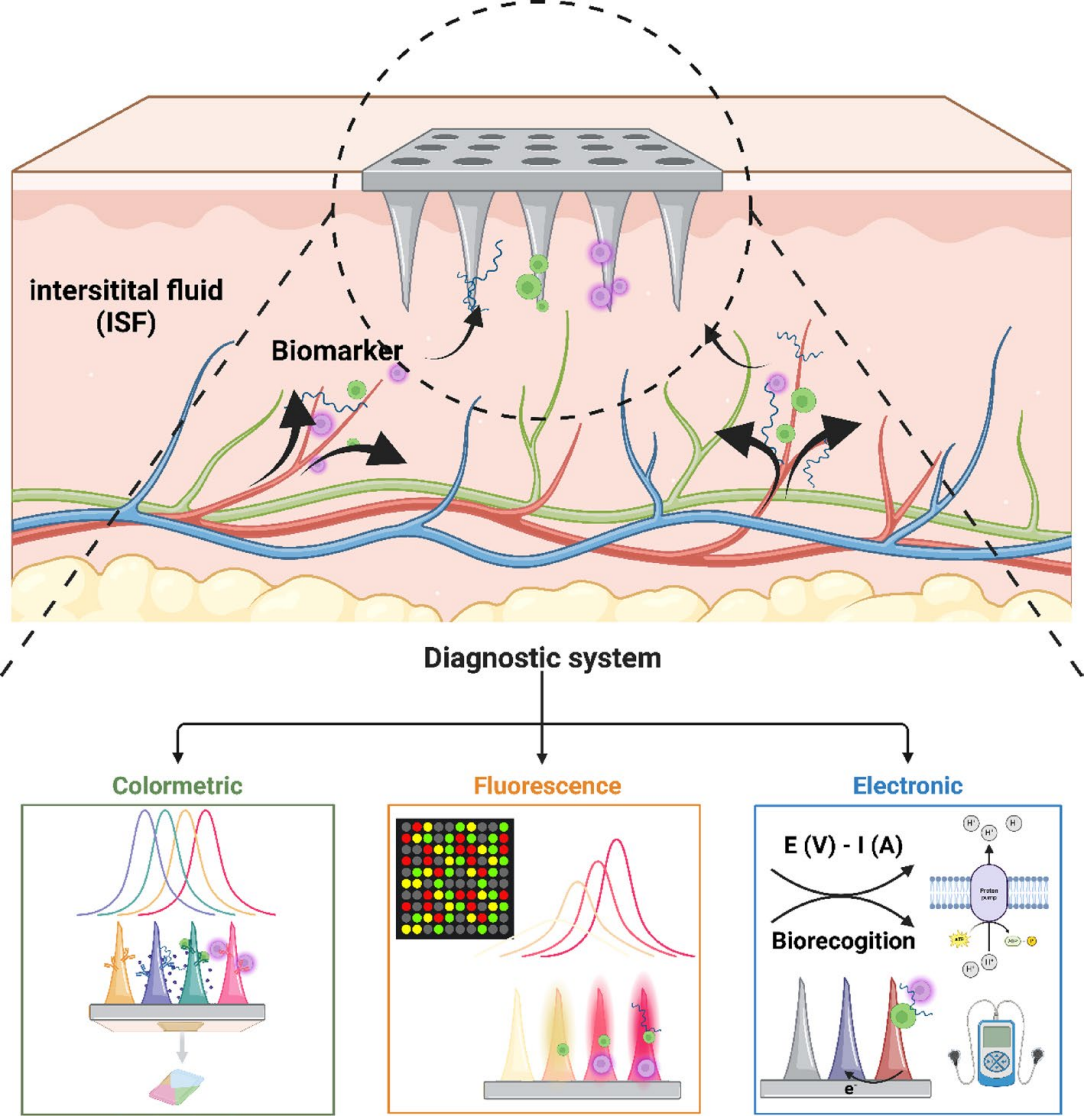
Schematic detailing metabolic syndrome diagnosis of interstitial fluid (ISF) using nanoparticles and microneedles. A microneedle penetrates the skin barrier to absorb ISF, and various nanoparticles supported on the microneedle detect biomarkers in ISF. Colorimetric, fluorescence, and electrochemical diagnostic methods are in the diagnosis system. Created with BioRender.com

Recent developments in point-of-care diagnostics have increased the significance of combining microneedles with nanoparticles to facilitate the extraction and measurement of metabolic syndrome-related biomarkers from interstitial fluid (ISF) in a timely, accurate, and minimally damaging manner[75]. Diagnostic tests have demonstrated increased sensitivity, specificity, and real-time monitoring capabilities based on recent comparative studies and performance evaluations, whereas earlier approaches just demonstrated proof-of-concept. According to recent research, cutting-edge glucose-responsive microneedle-nanoparticle platforms that employ phenylboronic acid derivatives or glucose oxidase (GOx) are more responsive and selective than conventional glucose detection techniques. These findings suggest potential advantages in clinical glucose monitoring. Continuous monitoring of clinically significant biomarkers, such as small molecules [76, 77], glucose [78–81], lactate [82, 83], cholesterol [84], inflammatory cytokines (such IL-6) [87], reactive oxygen species [85, 86]), and nucleic acids [87–90], has also been greatly facilitated by wearable microneedle diagnostics that employ biosensing nanoparticles. Recent clinical trials have demonstrated that, despite these encouraging results, there are still significant issues with repeatability, long-term stability, and signal interference that make practical application difficult. Disease diagnosis using colorimetric, fluorescence-based, and electrochemical detection techniques is illustrated in Fig. 3. A detailed analysis reveals that these detection techniques differ greatly from one another. This demonstrates the need for stronger validation in a variety of clinical scenarios, improved calibration standards, and signal amplification techniques. These crucial deficiencies must be addressed to bring microneedle-nanoparticle platforms closer to practical application.

Below, we describe recently reported microneedle nanoparticle-integrated diagnostic methods, their principles, and underlying mechanistic details. Table 4 lists representative studies summarizing them based on detection methods, target diseases, analysis time, and detection concentrations. The next sections explain the diagnostic technologies in greater mechanistic depth.

**Table 4:** Types of diagnostic and diagnostic efficiency of metabolic syndrome using nanoparticles and microneedles

Principles	Target biomarkers	Time consumption/continuous sensing	Detection ranges	Refs.
Colorimetric	Glucose	~ 10 min	~ 60.2 mg	[91]
Colorimetric	pH, glucose, other small molecules		6 < pH < 8, glucose > 2 mg/mL	[92]
Colorimetric	Glutathione (GSH)	~ 15 min	~ 0.36 μM	[93]
Fluorescence	IL-6	-	0.33 pg/mL	[94]
Fluorescence	Ferritin, folic acid (FA), and vitamin B12 (VB12)	~ 20 min	Ferritin ~ 0.035 ng/mL, HB FA 0.012 ng/mL, VB 0.029 ng/mL	[95]
Fluorescence	ISF endotoxin	~ 1 h	~ 0.0064 EU/mL	[96]
Electrochemical	Diabetes	~ 50 s	0.8–24 mM	[97]
Electrochemical	L-carnitine and choline	~ 10 min	–	[98]
Electrochemical	Cholesterol	~ 1 min	1–20 μM	[99]
Electrochemical	Alcohol	~ 35 s	0–80 mM	[100]
Electrochemical	Sodium	–	10–160 × 10⁻^3^ M	[76]
Electrochemical	Diabetic ketoacidosis (DKA)			[101]
Electrochemical	Glucose, uric acid, and cholesterol	~ 4 s	Glucose 2–12 mM Uric acid 0.1–1.2 mM Cholesterol 1–12 mM	[102]
Electrochemical	Lactate	~ 8 s	0–10 mM	[103]

### Colorimetric assay

Colorimetric detection is based on the idea that chemical or metabolic reactions might create products that have different optical absorption qualities. When an analyte reacts with an enzyme or catalyst, it either makes or changes chromogenic molecules that absorb visible light at certain wavelengths. The Beer–Lambert law says that the intensity of the color we see is directly related to the concentration of the analyte. This law connects absorbance to concentration and optical path length. This relationship makes it possible to make quantitative measurements by making calibration curves. Colorimetric assays are extensively used in point-of-care diagnostics because you can see the signal without needing complicated equipment [104, 105].

In comparison to traditional analytical procedures, colorimetric detection techniques offer advantages such as simplicity, low production costs, and rapid diagnostic capabilities [106, 107]. Recent research on metabolic disorders has demonstrated the ability of integrated colorimetric assays to simultaneously monitor many indicators, including insulin [108], glucose [109], uric acid [110], and pH [111]. A representative glucose-responsive colorimetric assay mechanism involves integrated glucose oxidase (GOx) within microneedles, which oxidizes glucose present in ISF into gluconic acid and hydrogen peroxide (H₂O₂). Subsequently, encapsulated horseradish peroxidase (HRP) nanoparticles catalyze the oxidation of 3,3',5,5'-tetramethylbenzidine (TMB) by H₂O₂, resulting in a visible blue color proportional to glucose concentration. This color change is quantified via imaging analysis software, demonstrating real-time and quantitative glucose detection capabilities. Gu et al. [79] developed a colorimetric microneedle patch utilizing GOx, HRP-loaded nanoparticles, and TMB, enhancing sensitivity via a dual-enzyme cascade reaction. Despite a thorough analysis indicating constraints regarding its practical use in complex physiological scenarios, this method exhibited rapid glucose detection within clinically significant concentration ranges. Inadequate attention was given to variations in ISF composition, potential interference from endogenous biomolecules, and stability concerns associated with enzyme-based systems. Moreover, there is a scarcity of comparisons with other advanced colorimetric microneedle systems utilizing nanoparticles functionalized with phenylboronic acid or metal–organic framework nanozymes. Future research must prioritize thorough validation and robust comparative evaluations to facilitate successful translation into clinical practice. Reproducibility, signal stability, calibration precision, and clinical efficacy across diverse physiological conditions must be prioritized.

**Fig. 4:**
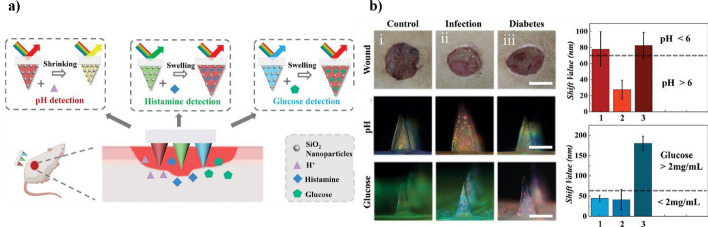
Structural color microneedle (EMN) patch for wound biomarker detection. **a** Schematic of pH-, histamine-, and glucose-responsive EMNs showing analyte-induced structural color shifts. **b** In vivo detection in control, infected, and diabetic wounds with corresponding spectral shifts. Thresholds indicate infection (pH > 6) and hyperglycemia (glucose > 2 mg/mL). This information has been reproduced with permission from Lu et al., Copyright (2023), Advanced Materials

Li and others made tiny needles from gold nanoparticles that can act like GOx to gather 60.2 mg of fluid from the skin in a lab in only 10 min, while also making glucose react to create H₂O₂ at the end of the needle. HRP trapped in the layer then helped to oxidize TMB using the H₂O₂ produced, resulting in OxTMB. HRP encapsulated in the layer catalyzed the oxidation of TMB, which was generated by the produced H₂O₂, to produce OxTMB. We observed colorimetric changes in the patches using different concentrations of glucose solutions (0–16 mM). As the glucose concentration increased, the patch colors darkened, allowing for quantitative analysis through RGB values. Hence, the colorimetric change between normal and hyperglycemic conditions could be observed using the naked eye, which was further quantified using ImageJ software. The skin simulation test using agarose gel clearly showed the change in patch color with different glucose levels (1, 5, 10, 15, and 20 mM). RGB analysis confirmed the linear relationship between the color and glucose concentration. When the patch was applied to diabetic and normal rats, it turned blue in diabetic rats, indicating high blood glucose, whereas no color change occurred in normal rats. The RGB value of the patch was consistent with the results of the blood glucometer. This proves that real-time glucose detection using the patch is possible and accurate. We can apply this technique as a diabetes diagnostic platform to overcome the high cost and ease of inactivating natural enzymes. Lu et al. investigated multiplexed screening of three wound inflammation-related biomarkers, including pH, glucose, and histamine, using silica nanoparticle-based photonic crystal microneedle arrays (Fig. 4a) [92]. Hydrogen ions react with carboxyl groups in hydrolyzed polyacrylamide to measure pH, while fluorophenylboronic acid is used to measure glucose. Histamine detection uses special molecules called aptamers that find histamine, and microneedle patches assist by changing color, which can be measured with light to see the color change and specific light pattern shifts in the photonic crystals (PhCs). The pH and glucose are also key biomarkers of metabolic diseases. Emerging microelectronic microneedles (EMNs) can detect pH through color changes and spectral shifts at different pH levels. In particular, a pH > 6 indicates the possibility of wound infection, in which case EMNs show a blueshift of approximately 70 nm or more, hence easily identifying infections. Hyperglycemia is characterized by a redshift of approximately 60 nm when the glucose concentration exceeds 2 mg/mL. To detect histamine, a key biomarker of wound inflammation, EMNs use a specific aptamer to bind histamine molecules. In tests with other similar compounds (like spermidine and tryptamine), EMNs only showed a noticeable change in the spectrum for histamine, proving they are very selective. We expect to use the developed microneedle array as a target for metabolic diseases. Zhao et al. developed swellable hydrogel microneedles composed of polyvinyl alcohol and sodium alginate using chemical cross-linking (polyvinyl alcohol/SA) and successfully extracted 6.4 mg of skin ISF in 15 min (Fig. 4b) [93]. A nanozyme (mixed valence cerium-metal organic frame: MCM) composed of an MnO₂-modified MCM was coupled to the reduction reaction of glutathione (GSH) with oxidized substrates. The optimal activity conditions for MCM were pH 4, 25 °C, and 10 mM TMB. The MCM nanozyme was able to identify various levels of GSH, ranging from 0.6 to 200 μg/mL, and could detect as little as 0.11 μg/mL (0.36 μM). In lab tests with agarose gels, when GSH was added, MCM stopped TMB from changing color, making the blue color fade away slowly. Detection performance was consistent for different GSH concentrations. Using the SD rat model, GSH was successfully detected in the skin interstitial fluid. The high GSH concentration, normal, and low GSH concentration groups all showed differences in GSH levels. Colorimetry confirmed the detection of GSH. Utilizing microneedle patches that employ such color changes is a painless and straightforward method for detecting other biomarkers that can indicate improved patient compliance [112]. While colorimetric microneedle assays offer notable benefits, they may occasionally produce semi-quantitative results, particularly when the color change is minimal. External factors such as ambient light or variations in skin surface can cause discrepancies, highlighting the necessity for built-in calibration steps. Studying methods to keep enzymes stable, like using nanoencapsulation or cross-linking, could improve their lifespan and dependability for long-term monitoring. Bringing together color-based detection with multiple sensing systems could improve diagnostic abilities, but it will require sophisticated image-processing algorithms to accurately understand complex color signals.

### Fluorescence assay

Fluorescence-based detection works by having specific molecules or nanomaterials take in light at a given wavelength of excitation and then release it at a longer wavelength of emission. When an analyte interacts with a fluorescent probe, it can change the strength of the emission, the wavelength of the light, or cause quenching effects. These changes in the signals make it very easy to see and react to the target. The strength of fluorescence is often linked to the analyte's concentration in a certain range of values. You can now find the concentration using calibration curves. Biomedical diagnostics are considerably more sensitive and can find a lot of things at once when you use sophisticated nanomaterials like quantum dots, dye-doped nanoparticles, and plasmon-enhanced fluorophores [113, 114].

**Fig. 5:**
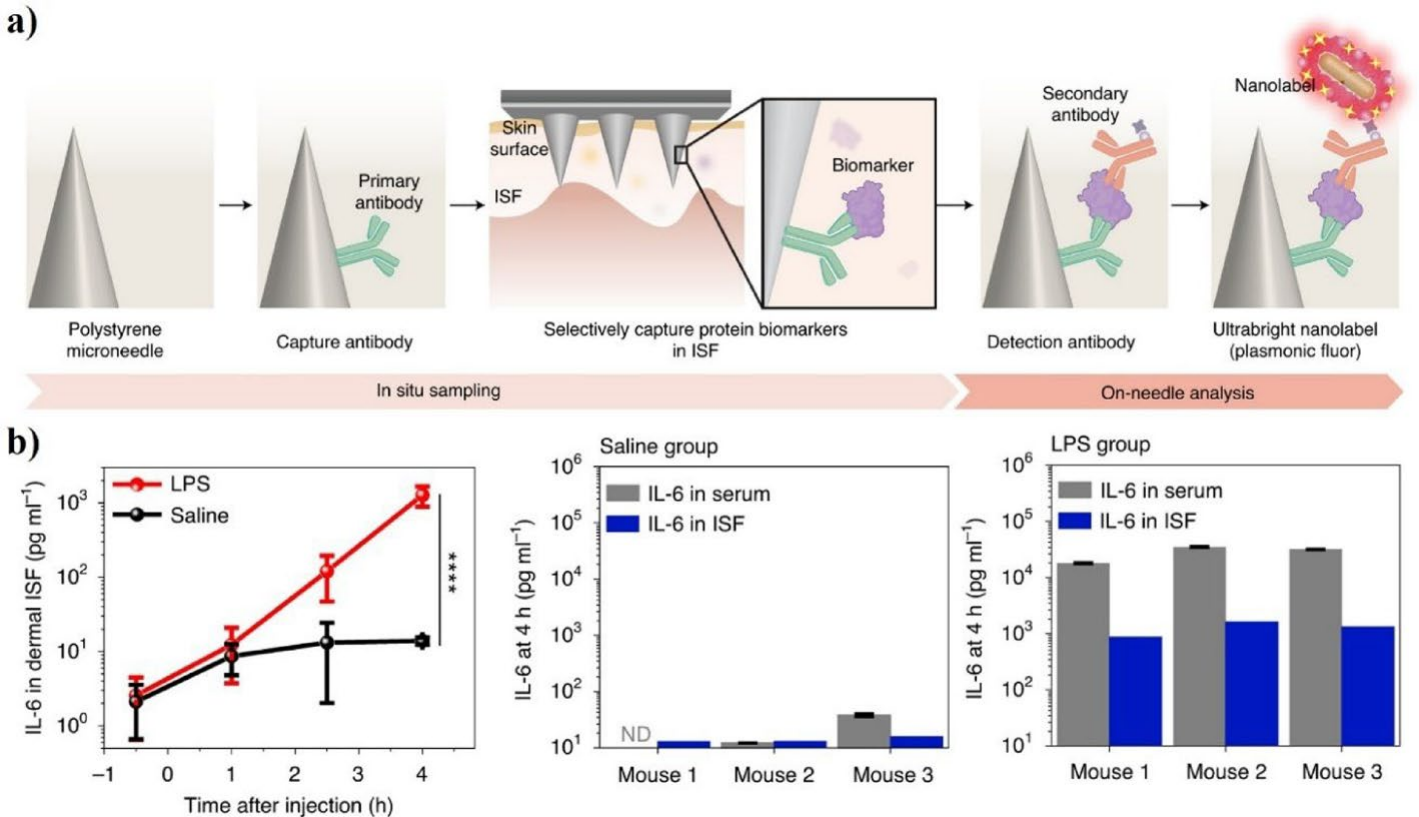
Ultrasensitive in situ detection of IL-6 in interstitial fluid (ISF) using a plasmonic fluor-based microneedle patch. **a** Schematic of the bilayer microneedle system for ISF protein biomarker quantification. Primary antibodies on polystyrene microneedles selectively capture IL-6 in situ, followed by on-needle sandwich immunoassay with a plasmonic nanolabel for signal amplification. **b** (Left) Time-course profile of IL-6 in ISF after LPS injection, showing rapid cytokine elevation versus saline control. (Right) IL-6 levels in ISF and serum at 4 h post-injection in individual mice, revealing lower absolute concentrations in ISF but consistent trends with serum profiles. Reproduced with permission from Yang et al., Copyright (2021), Nature Biomedical Engineering

Fluorescence detection is a highly sensitive technology for disease diagnosis, particularly for quantifying biomarkers [115–118]. This technique involves integrating photonic nanoparticles and microneedles, which can be used to create a sensitive and specific detection platform that utilizes a patient’s ISF for diagnosis [119–122]. In fluorescence-based microneedle assays, antibody-coated plasmonic nanoparticles are embedded into microneedles. Upon insertion into the skin, these nanoparticles selectively bind to specific biomarkers such as inflammatory cytokines (e.g., IL-6) present in ISF. When illuminated at specific wavelengths, the nanoparticles emit a fluorescent signal whose intensity correlates directly with the biomarker concentration, allowing ultrasensitive quantification. Such an approach enables rapid and precise monitoring of cytokine levels, facilitating effective inflammation management in metabolic disorders. This section explains a method that combines photonic nanoparticles and microneedles with microfluidic technologies to find signs of metabolic syndrome in interstitial fluid (ISF). In photonic-based detection systems, microneedles are primarily involved in capturing and concentrating biomarkers in the ISF. The capture process uses common detection methods like nucleic acid hybridization, antibody-antigen reactions, and antibody-target interactions. Photonic-based detection systems often use various expandable microneedles for ISF extraction. Wang Zheyu et al. presented a method for detecting and quantifying protein biomarkers in liver cell culture supernatant using microneedles patched with photonic nanoparticles (Fig. 5a) [94]. They used these microneedles, which had a detection limit approximately 800-fold lower than existing photonic dots, to detect various ISF protein biomarkers. They functionalized them with antibodies, detected them, and quantified them using fluorescence-enhanced immunological analysis. Mice were given lipopolysaccharide to trigger a quick immune response, and then microneedle patches were used to check the levels of interleukin-6, one of the inflammatory cytokines, over time. The results showed that the concentration of interleukin-6 in the ISF increased rapidly over time. At 0 h, a concentration of 2.6 ± 1.9 pg/mL was detected; after 4 h, the concentration was 1271.9 ± 393.4 pg/mL (Fig. 5b). Therefore, biomarkers can be detected in a rapid and non-invasive manner and analyzed; this is clinically promising for disease monitoring and vaccine effectiveness evaluations. Wu et al. reported a portable device based on microneedles attached with fluorescent labeling for the multiplex quantification of hemoglobin biomarkers [95]. This device used gold nanocubes covered with gold nanoparticles to boost the signals, making them thousands of times brighter than regular fluorescent markers. The developed device provides rapid and accurate hemoglobin quantification, allowing the simultaneous detection of major biomarkers such as hemoglobin, ferritin, folate, and vitamin B12. The device could detect hemoglobin in a range of 0.07 g/dL to 5.0 g/dL, ferritin at 0.035 ng/mL, folate at 0.012 ng/mL, and vitamin B12 at 0.029 ng/mL. It was also faster than existing commercial kits, with an analysis time of less than 20 min, and cost less than $2. In summary, using photonic nanoparticles and microneedles helps create a reliable and precise system for diagnosing diseases with ISF. Plasma samples from 37 patients were tested using a combination of special paper devices and enhanced fluorescence tests, showing that it can detect several biomarkers at the same time with outstanding sensitivity, specificity, and accuracy. Photobleaching and quenching affect fluorescence-based microneedle devices, resulting in compromised signal stability over time. Using outside photonic nanoparticles multiple times raises worries about possible immune reactions and long-term harm from ongoing use. Optimizing nanoparticle surface chemistry and using advanced filtering algorithms can reduce background fluorescence and enhance sensitivity. Additionally, large-scale manufacturing and standardized calibration procedures are essential for maintaining consistent performance among patient populations.

### Electrochemical assay

Electrochemical detection is based on the premise that biological reactions that happen at electrode surfaces create electrical signals, such as current, potential, or impedance, that can be used to figure out how much of an analyte is there. When a target analyte undergoes an oxidation–reduction (redox) process, the subsequent electron transfer is quantified and converted into an electrochemical response. Cyclic voltammetry (CV), differential pulse voltammetry (DPV), and chronoamperometry (CA) are some of the most prevalent approaches. Each of these approaches has a different amount of analytical sensitivity and range of motion. Faraday's law of electrolysis states that the magnitude of the signal we see is connected to the amount of charge that is transferred to the amount of analyte that is present. Electrochemical biosensors are becoming one of the most popular diagnostic tools for point-of-care use because they are exceedingly sensitive, respond rapidly, and operate with compact devices [123, 124].

Recent research has focused on biochemical sensors that provide personalized, user-friendly advantages [125]. These sensors can accurately detect low-molecule biomarkers owing to their rapid response, durability, and high sensitivity. They are composed of two parts: a recognition structure that captures analytes and identifies biological reactions, and a transformer structure that converts the captured analytes into electrical signals [126–128]. Electrochemical biosensors integrated with microneedles involve coating conductive polymer composites or enzyme-functionalized nanoparticles onto the microneedle surface. Target biomarkers (e.g., glucose) in ISF undergo enzymatic reactions at the electrode interface, causing electrochemical oxidation or reduction. This reaction generates measurable electrical signals proportional to biomarker concentration. Integrated electronic systems detect these signals, providing continuous, real-time monitoring of metabolic biomarkers with high precision and sensitivity. In this section, we provide a detailed introduction to microneedle- and nanoparticle-based electrochemical biochemical sensors. Electrochemical biochemical sensors are typically designed to detect various analytes using microfluidic systems. These include cyclic voltammetry, differential pulse voltammetry, square wave voltammetry, chronoamperometry, and current-based methods [129, 130]. Microfluidic platforms based on electrochemical sensors have been used to detect ions, such as K^+^ [77], Na^+^ [76], and pH [131], and low-molecular compounds, such as lactate [83], acetate [125], and glucose [81, 97, 132].

**Fig. 6:**
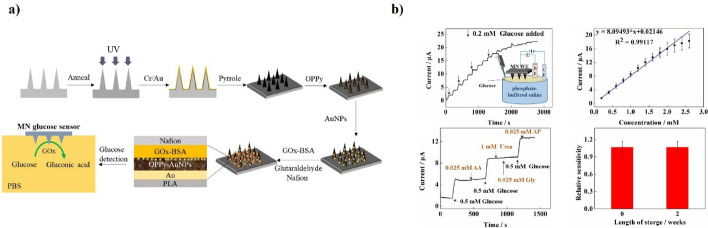
Fabrication and performance of a gold nanoparticle-deposited microneedle enzymatic glucose biosensor. **a** This schematic illustrates the stepwise fabrication process and multilayer structure of the microneedle (MN) sensor, which integrates gold nanoparticles (AuNPs) and enzyme immobilization for glucose detection. **b** The sensor exhibits a clear amperometric response to glucose, with a linear detection range up to 2.6 mM. It demonstrates high selectivity against common interferents (AA, urea, Gly, AP) and maintains stable sensitivity after 2 weeks of storage. Reproduced with permission from Liu et al., Copyright (2020), Electrochimica Acta

Recently, Zhang et al. reported a polylactic acid microbead-based glucose sensor for continuous glucose monitoring [133]. The sensor used gold and oxidized polyphenols to help the polylactic acid microneedles conduct electricity, and it had a mix of gold nanoparticles, GOx, and Nafion film in the microbead electrode to measure glucose levels. The glucose sensor showed an electrical conductivity of 8.09 μA/mM, could detect glucose levels as low as 40 μM, and worked well for glucose concentrations between 0 and 2.6 mM. Similarly, Ghavami Nejad et al. reported the development of a polymer-based hydrogel sensor for real-time and non-enzymatic glucose detection in ISF using poly(3,4-ethylenedioxythiophene) polystyrene sulfonate and silver-platinum nanoparticles (Fig. 6a) [134]. The hydrogel sensor used platinum nanoparticles, made by changing platinum ions into solid particles through a reaction with dopamine and silver nanoparticles, to help detect glucose. The hydrogel sensor demonstrated high sensitivity (0.9 mM) and successfully monitored glucose levels in a type 1 diabetic mouse model in real time. Researchers have also explored the use of electrochemical biosensors for detecting various biomarkers (Fig. 6b).

Mugo et al. reported a multiple electrochemical microbiosensor based on a PDMS/carbon nanotube (CNT)/cellulose nanocrystal (CNC) composite (PDMS/CNT/CNC@PDMS), capable of detecting pH, epinephrine, dopamine, and lactate on a per-layer basis [135]. The pH sensor was created using a layer that mixed pH-sensitive polyaniline (PANI), carbon nanotubes (CNT), cellulose nanocrystals (CNC), and silver nanoparticles, while the sensors for epinephrine, dopamine, and lactate had that same layer plus an additional layer adjusted with either epinephrine, dopamine, or lactate along with PANI-co-3-aminobenzoic acid and gold nanoparticles. These sensors were able to find specific substances quickly (in about 2 min) and could detect very small amounts: 0.0007 ± 0.0002 μM for epinephrine, 2.11 ± 0.05 nM for dopamine, and 0.07 ± 0.07 mM for lactate. The pH sensor accurately detected a pH range of 4.25–10. Therefore, electrochemical biosensors integrated with micro- and nanoparticles offer the advantage of easy signal generation and detection, making them ideal for long-term monitoring. However, more research is needed to create a complete electrochemical biosensor that can accurately detect many different metabolic syndrome markers in interstitial fluid. To ensure stable long-term performance, it is essential to tackle potential electrode fouling and drift, as these factors can undermine measurement accuracy over time. Accurate readings across various analyte concentrations rely on robust reference electrode design and the reduction of interference paths. Research on novel nanocomposites and advanced materials, such as conductive polymers, may enhance sensitivity and biocompatibility. The transition of electrochemical microneedle sensors from laboratory prototypes to practical clinical devices hinges on the development of standardized calibration methods and scalable manufacturing processes. Table 5 lists recent research that has used nanoparticle-integrated microneedle systems in both the diagnostic and therapeutic domains to put these developments in even more context. The sector is seeking a variety of design approaches, nanomaterial kinds, and therapeutic goals, as demonstrated by these illustrative instances.

**Table 5:** Recent primary research on nanoparticle–microneedle systems

Study (year)	MN type	Nanoparticle type	Application	Key findings	Refs.
Gao et al. (2024)	Dissolving + Photothermal	Polydopamine NPs	Obesity	WAT browning, ~ 19% body weight reduction in obese mice	[67]
Lin et al. (2024)	Composite swellable MN	Insulin NPs	Diabetes	Glucose-responsive insulin release, prolonged glycemic control	[136]
Liu et al. (2024)	Hollow + Sensor-integrated	PEGylated insulin NPs	Diabetes	Real-time glucose monitoring and responsive insulin delivery in vivo	[137]
Shen et al. (2024)	Dissolving	Calcium peroxide NPs	Diabetic wound healing	Sustained O₂ release, biofilm inhibition, accelerated wound closure in diabetic models	[138]
Zhao et al. (2025)	Hydrogel-forming	HA hydrogel NPs	Wound healing	Improved skin repair via sustained localized delivery	[139]

Recent advancements in miniaturized and wearable electrochemical biosensors have significantly improved the real-time monitoring of metabolic syndrome markers, including glucose, cholesterol, and inflammatory cytokines such as IL-6 and TNF-α [140]. Despite substantial progress, including the development of non-enzymatic glucose sensors employing advanced heterostructures and versatile platforms for multiplexed cytokine detection, several critical challenges remain [141, 142]. This involves ensuring the enduring stability of biosensors, minimizing biofouling effects, and alleviating any signal interference during continuous monitoring. Furthermore, the conversion of these technologies from laboratory prototypes to clinically reliable systems require comprehensive evaluation of sensor accuracy, reliability, and biocompatibility under diverse physiological conditions. Addressing these problems is essential for fully realizing the clinical potential of wearable biosensors in comprehensive metabolic health assessment.

## Treatment

**Fig. 7:**
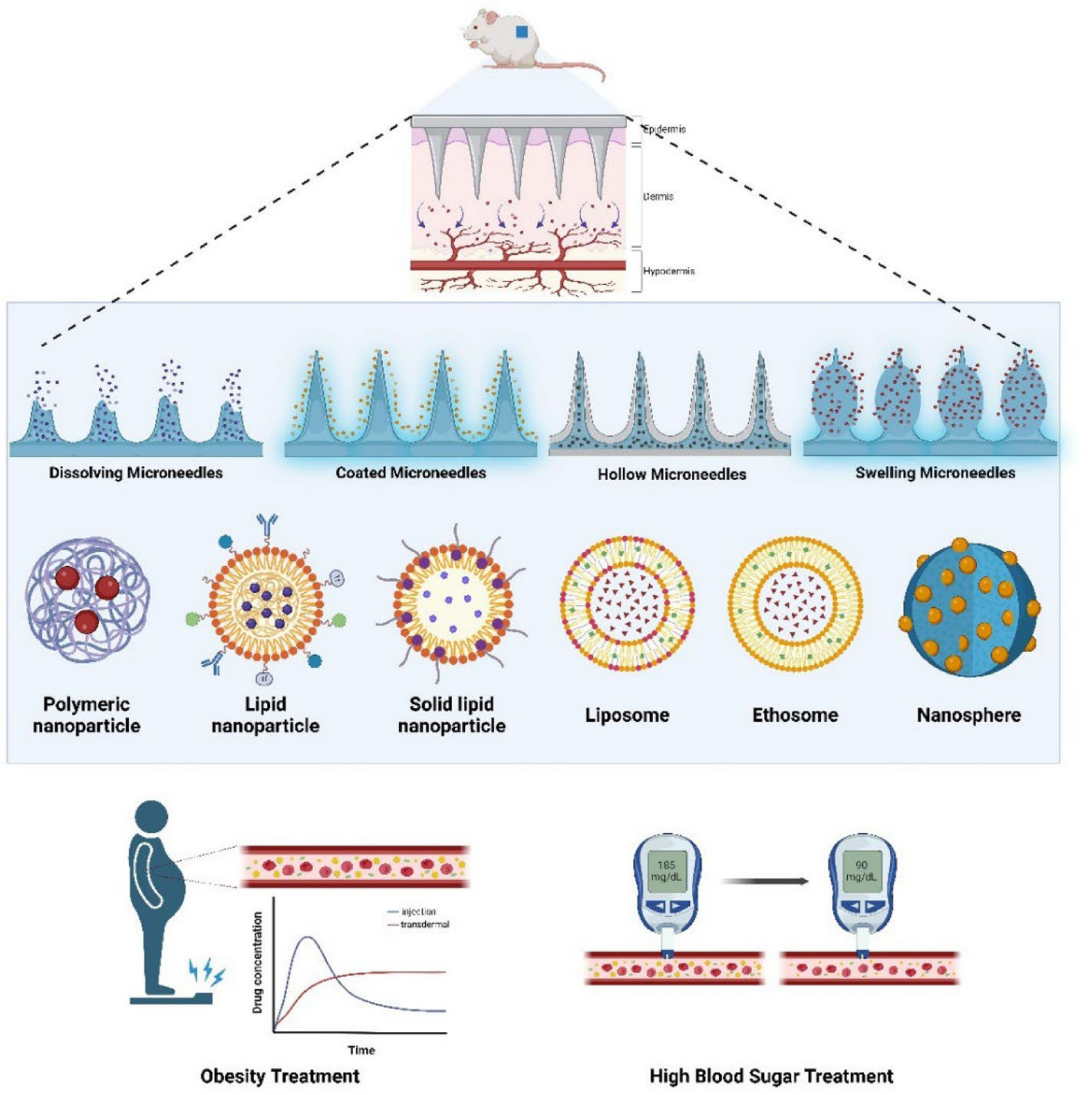
Schematic of metabolic disease treatment using various microneedle types and nanoparticles. Nanoparticle-loaded drugs are applied to microneedles for sustained delivery within the skin dermis layer. Microneedle types include dissolving, coated, hollow, and swelling microneedles. Created with BioRender.com

Unlike in the past, people in modern society are at an increased risk of developing various metabolic diseases because of their exposure to various foods. Therefore, the incidence of metabolic diseases has steadily increased, necessitating new and more effective therapeutic methods to treat these conditions. Therapeutic methods utilizing nanoparticle technology are an emerging area in medicine and biotechnology. Nanoparticle technology involves the design, manipulation, production, and application of materials with a diameter < 100 nm. It has numerous medical applications, including drug delivery systems, diagnostics, gene delivery systems, and scaffolds for tissue engineering [143–146]. Nanoparticle-based drug delivery systems for metabolic disease treatments still require improved drug efficiency and enhanced convenience of administration for patients [147, 148]. Research is underway to integrate microneedle technology with nanoparticle technology to address these issues (Fig. 7) [149]. The integration of this technology is expected to enhance both efficiency and biocompatibility. To comprehend the therapeutic and diagnostic impacts of microneedle systems that use nanomaterials, it is vital to know how cells and molecules govern them, as well as how drugs are released from them. After transdermal delivery, nanoparticles released from microneedles are absorbed by skin-resident cells, including keratinocytes, fibroblasts, Langerhans cells, and macrophages. Uptake transpires by many mechanisms, including clathrin-mediated endocytosis, caveolae-dependent endocytosis, phagocytosis by immune cells, and, at times, direct membrane fusion, contingent upon surface chemistry and particle size [150, 151]. Nanoparticles that have been taken in can either avoid being broken down by endosomes or be broken down by lysosomes. This decides where therapeutic payloads go, either to the cytosol or to start localized immune responses. Nanoparticle cargo also starts signal transduction cascades that change how cells respond to things. Polymeric or lipid nanoparticles that transport metabolic medicines have been demonstrated to activate AMPK pathways, facilitating cellular glucose uptake and fat catabolism [152]. Antioxidant nanomaterials can reduce the amount of ROS in cells. This lessens oxidative stress and changes pathways like NF-κB, which lowers the production of pro-inflammatory cytokines [153]. These intracellular reactions demonstrate that the therapeutic effects are contingent not only on the rate of nanomaterial release but also on the subsequent molecular signaling.

### Mechanistic insights

Microneedle-nanoparticle integrated systems provide controlled drug release through precise modulation of nanoparticle attributes, including size, surface charge, and biodegradability, thereby providing sustained therapeutic administration via encapsulation within the nanoparticle matrix. Post-microneedle insertion, nanoparticles swiftly infiltrate and distribute inside dermal tissues via temporary microchannels formed by the microneedles, with smaller nanoparticles (< 100 nm) exhibiting superior dispersion into deeper strata. Furthermore, intradermally administered nanoparticles interact intimately with dermal immune cells, such as dendritic cells and macrophages, thereby modulating local immune responses and reducing systemic exposure, which enhances therapeutic efficacy, biocompatibility, and patient safety. Recently in vivo pharmacokinetic investigations validated consistent localized concentrations and extended therapeutic effects, highlighting the potential for successful transdermal treatments in managing metabolic diseases.

**Fig. 8:**
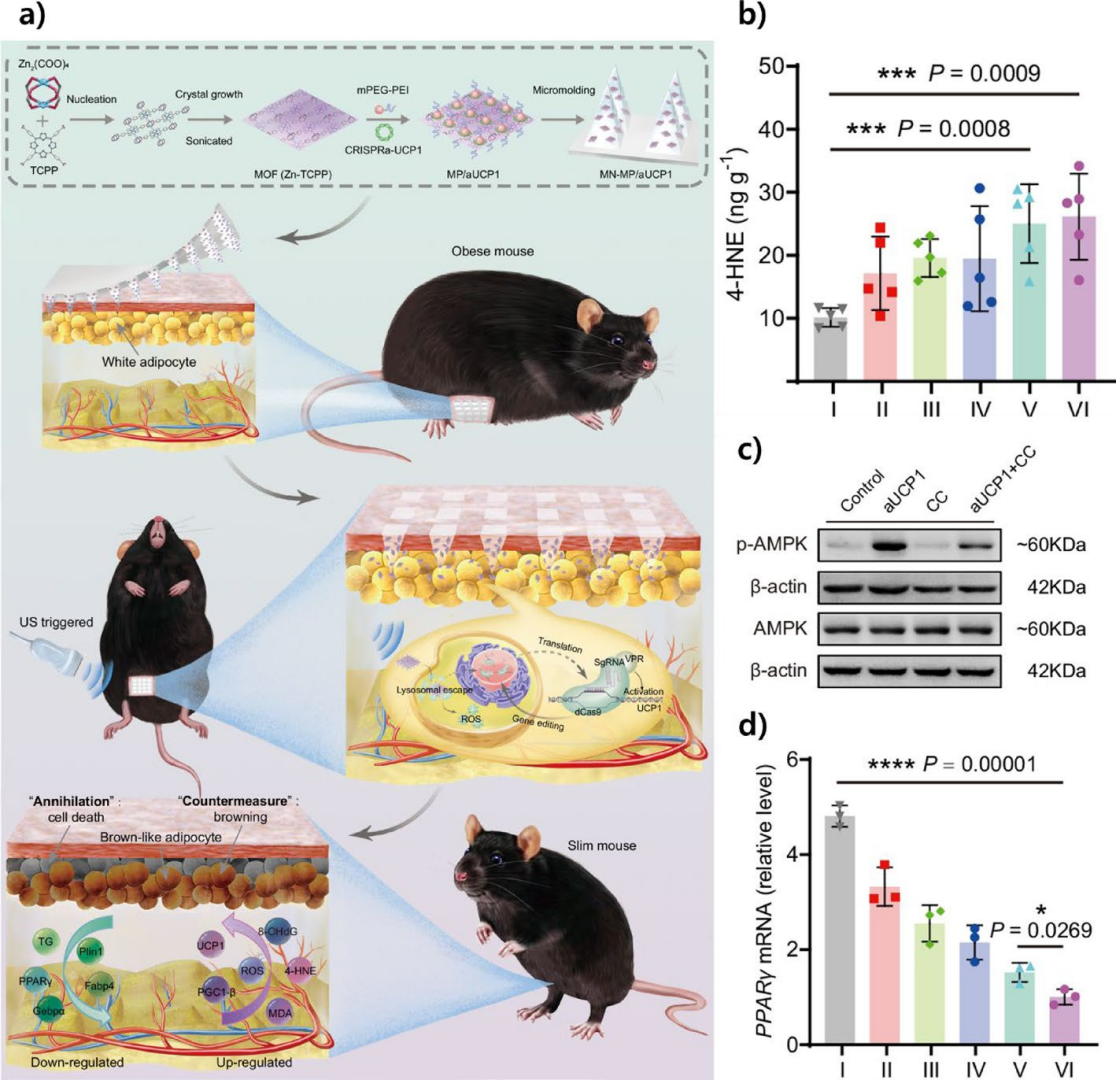
Microneedle-assisted, ultrasound-triggered CRISPRa therapy enhances UCP1 expression, activates AMPK signaling, and reduces PPARγ-driven adipogenesis. **a** Schematic overview of MOF-based CRISPRa delivery via microneedle patches. **b** Reduced 4-HNE levels indicate suppression of oxidative stress. **c** Western blot confirms AMPK activation. **d** qPCR shows decreased PPARγ mRNA levels after treatment. Reproduced with permission from Li et al., Copyright (2025), Nature Communications

Microneedle–nanoparticle integrated systems offer a flexible method for delivering treatments by using small molecular processes to get specific biological effects. These systems enable the precise spatial and temporal delivery of functional biomolecules—such as nucleic acids, antioxidants, and anti-inflammatory agents—via transdermal routes, minimizing systemic exposure and maximizing local efficacy. One prominent mechanism involves gene modulation using nanoparticle carriers. As illustrated in Fig. 8, microneedle patches have been developed to deliver metal–organic framework (MOF)-based CRISPR activation (CRISPRa) systems targeting adipocytes. When the microneedle patches are used, the CRISPRa components raise the amount of uncoupling protein 1 (UCP1), which enhances mitochondrial function and helps change white fat into brown fat. This activation is accompanied by phosphorylation of AMPK and suppression of PPARγ expression, ultimately reducing lipid accumulation and contributing to weight loss [154].

**Fig. 9:**
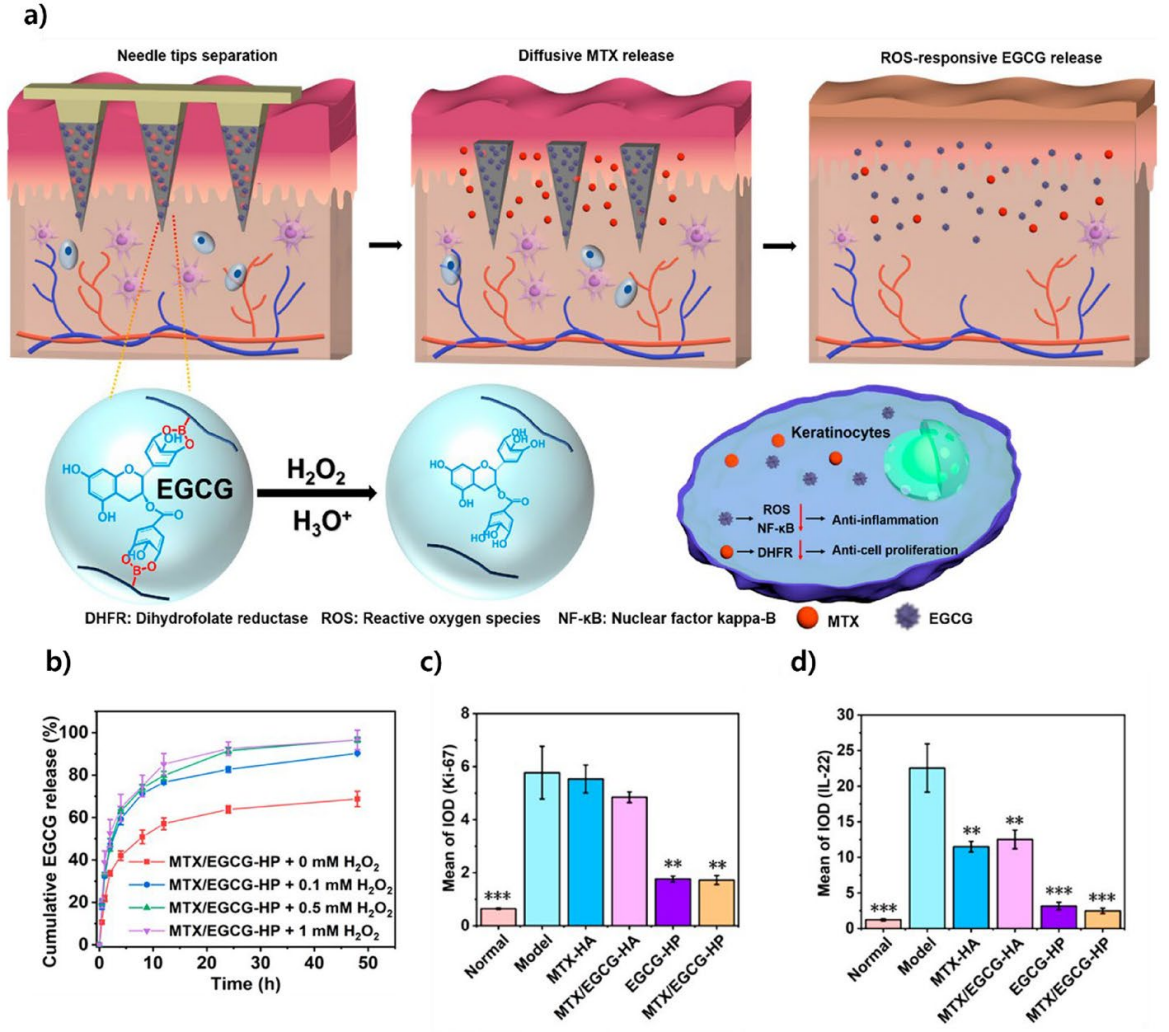
The ROS-responsive MN patch's therapeutic mechanism and efficacy are examined. **a** Schematic of MN-mediated dual drug release: rapid MTX release and ROS-triggered EGCG release. **b** Cumulative EGCG release increases with H₂O₂ concentration, confirming ROS-responsiveness. **c** Ki-67 expression significantly decreased in MN@MTX/EGCG-treated skin, indicating reduced proliferation. **d** IL-22 levels markedly suppressed by EGCG-containing MNs, showing strong anti-inflammatory effects. Reproduced with permission from Bi et al., Copyright (2023), ACS Nano

Another important pathway involves the mitigation of oxidative stress through ROS-responsive delivery. Figure 9 presents a dual-release microneedle platform that incorporates methotrexate (MTX) and epigallocatechin gallate (EGCG). The microneedles are designed to release MTX rapidly and EGCG in response to elevated ROS levels. TThis timed release helps reduce the rapid growth of skin cells and the inflammatory substance IL-22, which aids in calming down psoriasis-related skin inflammation.

**Fig. 10:**
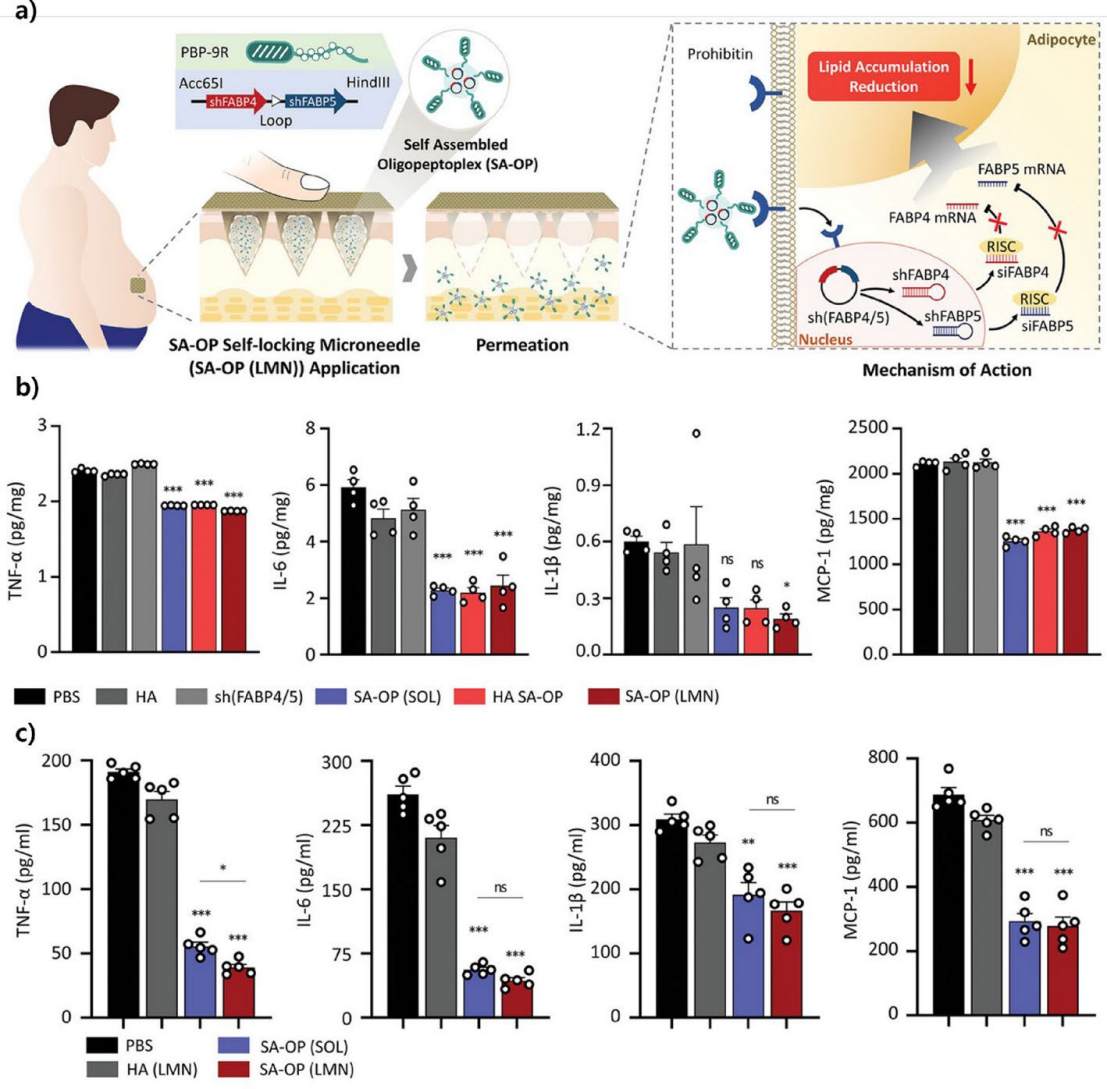
Anti-inflammatory mechanism and cytokine-suppressing effects of SA-OP-loaded microneedle patches. **a** Schematic illustration of the self-assembled oligopeptoplex (SA-OP) microneedle (LMN) system. After being applied to the skin, LMNs dissolve and release SA-OPs, which move into fat cells and help silence FABP4 and FABP5 through the RNA interference pathway, resulting in less fat buildup and inflammation. **b** The levels of cytokine expression in differentiated adipocytes were measured in vitro after treatment. SA-OP(LMN) significantly suppressed pro-inflammatory cytokines TNF-α, IL-6, IL-1β, and MCP-1 compared to controls. **c** The study measured in vivo serum cytokine levels in mice that were induced to be obese by a high-fat diet. SA-OP(LMN) markedly reduced TNF-α, IL-6, IL-1β, and MCP-1 levels, confirming systemic anti-inflammatory effects. Reproduced with permission from Choi et al., Copyright (2024), Advanced Materials

In addition to localized therapeutic effects, microneedle platforms have demonstrated systemic immunomodulatory potential. Figure 10 shows that dissolvable microneedles filled with self-assembled oligopeptoplexes (SA-OPs) can effectively deliver siRNA treatments into fat cells. This results in lower amounts of FABP4 and FABP5, which greatly reduces pro-inflammatory substances such as TNF-α, IL-6, IL-1β, and MCP-1 in both laboratory experiments and living beings. These cytokine-suppressing effects highlight the capacity of microneedle systems to address chronic inflammation associated with metabolic diseases [155].

Collectively, these examples underscore the versatility of microneedle–nanoparticle platforms in targeting complex metabolic pathways. By activating genes, delivering drugs that respond to changes in the body, and adjusting cytokines, these systems allow for customized treatment plans that match the complicated nature of metabolic syndrome. To show how these methods can be applied in real life, Table 6 lists recent clinical trials and patents related to microneedle–nanoparticle systems for treating metabolic diseases. These entries reflect the growing clinical and commercial interest in leveraging such integrated platforms for diagnostics, therapeutics, and combination strategies.

**Table 6:** Summary of recent clinical trials and patents related to microneedle–nanoparticle platforms for metabolic syndrome

Field	Title	Date	MNs type	Disease	Target/administered medication	NCT number
Diagnosis	Glucose Measurement Using Microneedle Patches (GUMP)	12/2017–01/2018	Biocompatible polymers or metal	Diabetes	Glucose	NCT02682056
Treatment	Enhanced Epidermal Antigen-Specific Immunotherapy Trial -1 (EE-ASI-1)	09/2016–12/2019	–	Type 1 diabetes	Gold particle-peptide (Insulin)	NCT02837094
Treatment	Multi-day (3) In-patient Evaluation of Intradermal Versus Subcutaneous Basal and Bolus Insulin Infusion	02/2012–05/2012	–	Type 1 Diabetes	Insulin	NCT01557907
Treatment	Insulin Delivery Using Microneedles in Type 1 Diabetes	2009/02/04–2014/01/08	Hollow microneedles	Type 1 Diabetes	Insulin	NCT00837512
Treatment	Adalimumab Microneedles in Healthy Volunteers	2018/07/11–2018/10/30	Hollow pyramid-shaped microneedles	Auto-immune/Auto-inflammatory	Adalimumab SC	NCT03607903
Treatment	A Pilot Study to Assess the Safety, PK, and PD of Insulin Injected Via MicronJet or Conventional Needle (MicronJet)	2008/03–2009/07	MicroJet	Type 2 diabetes	Insulin	NCT00602914
treatment	Glucose-responsive insulin delivery microneedle system	2021–12-07	Hypoxia-sensitive hyaluronic acid (HS-HA)	Diabetes	Insulin	US11191815B2
treatment	Enhanced cancer immunotherapy by microneedle patch-assisted delivery	2022–02-16	pH within the acid-degradable nanoparticles	Cancer	Anti-PDl antibody	EP3804620B1
Diagnosis	Biosensing device	2018–05-04	Metal nanoparticle (165d)	diabetes	Glucose	KR101855579B1
Treatment	Polypeptide nanoparticle for treating diabetes, polypeptide nanoparticle microneedle, and preparation method thereof	2019–11-15	Carboxymethylcellulose/Hyaluronic acid	Type 2 diabetes	Polypeptide drug	CN111053891A
Treatment	Glucose-responsive insulin delivery microneedle system	2021–12-03	Glucose-responsive insulin	Type 1 Diabetes	Insulin	US20220160841A1
Diagnosis	In Vivo Extraction of Interstitial Fluid Using Hollow Microneedles	2019–02-25	Extraction of interstitial fluid using hollow microneedles	Metabolic stress and fatigue	Lactate	US20190274599A1

Table 7 presents a comparative overview of recent experimental studies employing microneedle–nanoparticle hybrid platforms across various disease categories, including metabolic disorders, skin diseases, and neurological conditions. Each case is categorized by microneedle type, nanoparticle composition, therapeutic or diagnostic strategy, and observed efficacy. The table highlights the versatility of hybrid platforms in achieving localized delivery, sustained release, and enhanced biosensing capabilities. Notably, dissolvable microneedles incorporating responsive nanoparticles have demonstrated promising results in glucose monitoring and gene therapy for obesity, while coated or hydrogel-based systems have been employed for wound healing and immunomodulation. This comparative analysis underscores the translational potential of tailored microneedle–nanoparticle systems in disease-specific contexts.

**Table 7:** Comparative Analysis of Transdermal Therapeutic Technologies

Characteristics	Conventional transdermal patch	Traditional microneedle systems	MN–NP hybrid systems	Refs.
Drug loading capacity	Moderate	Moderate	High	[156]
Precision in therapeutic delivery	Low	Moderate to high	Very high	[157]
Patient compliance	High	High	Very high	[158]
Controlled drug release	Limited	Good	Excellent	[159]
Immune reaction risk	Low	Low to moderate	Low	[160]
Biodegradability	Variable	Moderate	Excellent	[161]
Manufacturing scalability	High	Moderate	High	[162]

### Basic metabolic disease treatment methods.

**Fig. 11:**
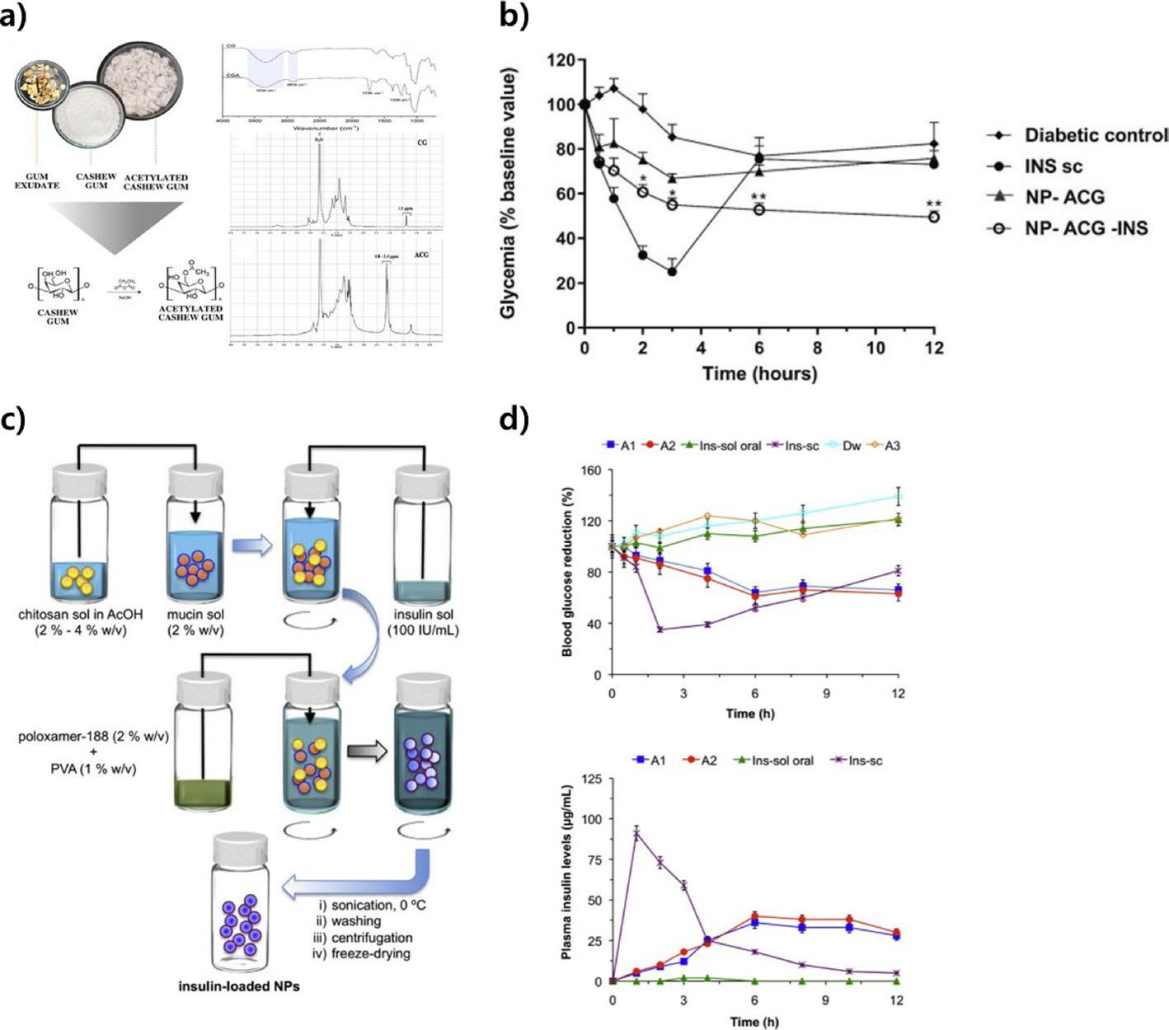
Orally administered insulin-loaded nanoparticles for treating diabetes. **a** Chemical structure and FTIR spectra for the CG acetylation reaction. The mechanism of insulin release from nanoparticles at pH 1.2 and 6.8 is also discussed. **b** Measurement of glycemic changes after a single oral dose, diabetic control, INS (4 IU/kg) SC, INS-ACG (50 IU/kg), and insulin-loaded nanoparticles (NP-ACG-INS). Data are expressed as mean ± epm (n = 6/group). Reproduced with permission from. Copyright (2022) Elsevier. **c** Schematic diagram of preparing insulin-loaded nanoparticles using double emulsion. **d** Percentage of blood glucose and plasma insulin levels in diabetic mouse models according to insulin dosing regimen. Abbreviations: ins-sol oral, insulin solution; ins-sc, insulin injection; A1, A2, insulin-loaded NPs containing 2 w/v% chitosan; A3, insulin-unloaded NPs. Data are presented as mean ± SD (n = 5). Reproduced with permission from. Copyright (2020) Elsevier

Researchers are developing most glucose-lowering agents for oral administration to improve glycemic control in patients with type 2 diabetes. Oral insulin administration can provide high glucose-enhancing properties because it mimics the body’s normal insulin pathway in a manner that is easily accessible to patients [163–165]. However, the short lifespan of the drug in gastric and intestinal fluids, which activate protein degradation, complicates ensuring high efficiency [166]. Therefore, researchers are continuing to develop blood sugar regulators that combine nanoparticle technology with insulin to address this challenge. In a recent study, Eliadna de Lemos Vasconcelos Silva et al. developed nanoparticles for orally administering insulin based on acetylated cashew gum (ACG) and chitosan (Fig. 11a) [147]. Cytotoxicity assays were performed on the HT-29 cell line to verify biocompatibility. TThe effect of lowering blood sugar was tested in male Wistar rats to compare how long the commercially available subcutaneous insulin injection and the insulin-loaded nanoparticles (NP-ACG-INS) worked.The results showed that the subcutaneous insulin injection provided the highest glycemic lowering, but with a duration of 2–3 h, whereas the fabricated NP-ACG-INS had a duration of approximately 12 h and a sustained reduction in blood glucose to 51.0% of baseline levels after 12 h (Fig. 11b). Momoh A. Mumuni and David Díaz et al. developed insulin-loaded mucosal adhesive nanoparticles based on the mucus-chitosan complex for oral drug delivery for sustained insulin release at the absorption site through mucus adhesive properties and tight functionalization via chitosan (Fig. 11c) [167]. The prepared insulin-loaded nanoparticles were evaluated for their encapsulation efficiency and loading capacity to confirm their insulin-loading ability. Four experimental groups—insulin administered via subcutaneous injection (ins-sc), insulin solution, insulin-loaded nanoparticles containing 2 w/v% chitosan, and insulin-unloaded nanoparticles—were compared to determine their antihyperglycemic effects in the diabetic albino Wistar mouse model. The mouse model treated with INS-SC showed a blood glucose lowering effect of up to 68% within 4 h; however, it was short-lived and increased to almost 100% of the initial level within 6–8 h. Conversely, the mouse model treated with insulin-loaded nanoparticles showed reduced blood glucose levels; the blood glucose-lowering effect lasted up to 12 h compared to ins-sc (Fig. 11d).

These results demonstrate that insulin integrated with nanoparticle technology has demonstrated high stability as a drug for treating metabolic diseases such as type 2 diabetes and has a longer duration and higher efficiency than conventional orally administered insulin. However, oral drug delivery typically travels through the digestive tract. Most of the drug is absorbed in the liver and small intestine. Hence, less drug is absorbed than is administered, and oral drug delivery is characterized by low efficiency and a long time to lower blood glucose in patients with hyperglycemia. These inherent features of oral administration necessitate the introduction of new treatment modalities. Additionally, nanoparticle encapsulation does not fully prevent enzymatic degradation and first-pass metabolism, highlighting the necessity for advanced protective coatings or pH-responsive materials. Strategies like ligand-mediated uptake or intestinal permeation enhancers can enhance gut absorption. Moreover, extensive stability tests and cost analyses are essential prior to the widespread adoption of these innovative formulations. In the future, combining oral nanoparticle methods with other drug delivery systems like microneedles could offer better ways to manage blood sugar levels.

### Transdermal drug delivery system

**Fig. 12:**
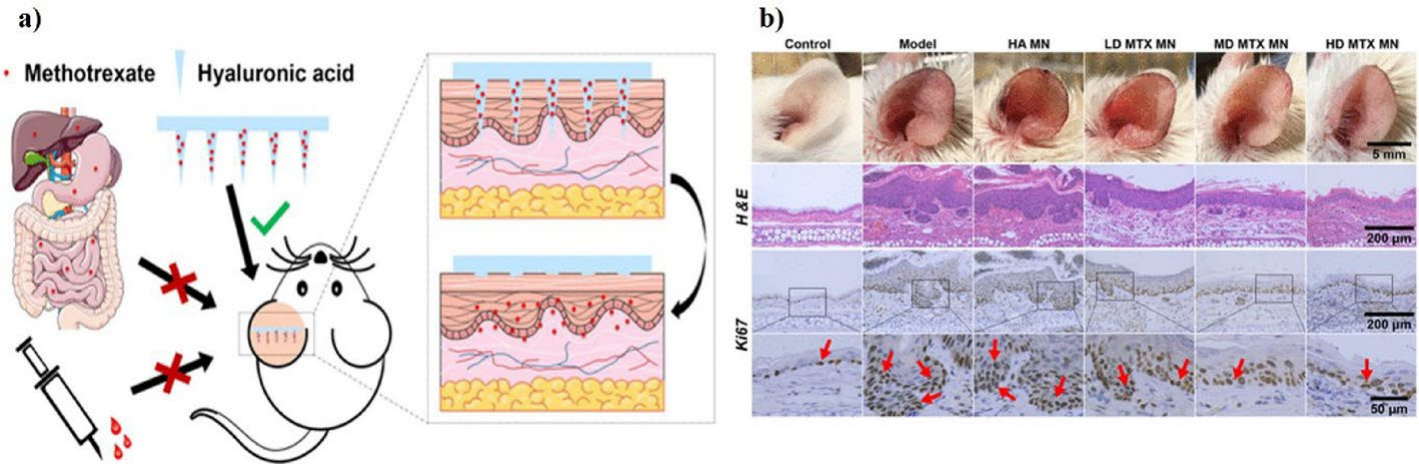
Methotrexate (MTX)-Loaded Hyaluronic Acid Microneedles for the Treatment of Psoriasis. **a** Schematic diagram of the fabrication of hyaluronic acid-based microneedles loaded with MTX to improve the treatment of psoriasis. **b** In vivo effects after application of MNs loaded with different concentrations of MTX in a mouse model with psoriasiform dermatitis, H&E staining, and changes in thickness of the ears according to treatment were measured. Reproduced with permission from. Copyright (2019) ACS Publications

Recently, several pioneering studies have been conducted using microneedles to deliver drugs incorporated with nanoparticles to treat metabolic diseases safely and easily compared to treatment using subcutaneous injections. A study by Xing-Qun Pu and Xiao-Jie et al. suggests that integrating nanoparticle-loaded drugs with microneedles can broaden the scope of transdermal drug delivery via polymeric microneedles because nanoparticles can effectively deliver both hydrophilic and hydrophobic drugs [168]. Nanoparticles can create an even spread of medicine inside the microneedles, which helps keep the medicine stable and works better together than regular drug delivery methods. These advantages of integrating nanoparticles and microneedles can be utilized to develop therapeutic agents for metabolic diseases. The types of microneedles discussed in Sect. 2 are chosen based on the application and purpose. Dissolving, swelling, and coated microneedles are made from highly biocompatible, low-cost, water-soluble, or degradable polymers, including hyaluronic acid (HA) (Fig. 12a) [169], gelatin (Fig. 12b) [170], silk fibrin [171], and polyvinylpyrrolidone [172]. These biodegradable microneedles dissolve in the skin dermis and release the encapsulated drug, which ensures high stability. This technology is particularly beneficial for patients with metabolic diseases who require continuous management. Numerous studies are currently ongoing on integrating drug-loaded nanoparticles with microneedles.

Recently, therapeutic microneedles have been developed with drugs incorporated into their internal structure to ensure controlled and effective drug release. This design enhances drug stability and offers significant advantages for therapeutic microneedle production and distribution. Representative research cases are summarized in Table 8, which includes information on the types of microneedles used, material types, nanoparticle types, target diseases, drug types, and drug release times. These microneedle platforms effectively deliver hydrophilic and hydrophobic agents, but optimizing their mechanical integrity is essential for reliable skin penetration. Integrating nanoparticles into the microneedle matrix necessitates attention to payload compatibility and release kinetics. Advanced manufacturing techniques like UV cross-linking and 3D printing help ensure uniform needle geometry and effective drug loading. Exploring stimuli-responsive or environmentally triggered microneedles could enable personalized dosing regimens. In vivo studies that compare different combinations of polymers and nanoparticles will help doctors and researchers pick the best methods for treating specific metabolic disorders.

**Table 8:** Material-dependent characterization and drug efficacy analysis of microneedles and nanoparticles for treating metabolic diseases

Types of microneedles used	Material types	Nanoparticle types	Target disease	Drug types	Drug release times	Mechanism	Refs.
Dissolving microneedles	Polyvinyl alcohol, Polyvinylpyrrolidone	Solid lipid nanoparticles	Lymphatic filariasis	Doxycycline monohydrate Albendzole Diethylcarbamazine	MNs ~ 72 h Oral ~ 24 h	Diffusion-controlled release from liquid matrix	[173]
Coated microneedles	Cellulose	Lipid-coated Cisplatin nanoparticles	Head and neck cancel	Cisplatin	~ 72 h	Dissolution of MN releases surface-coated NPs, followed by diffusion of drug	[22]
Swelling microneedles	Hyaluronic acid	Human serum albumin nanoparticles	Psoriasis	Methotrexate	~ 48 h	Swelling-induced diffusion of drug	[21]
Dissolving microneedles	Hyaluronic acid	Mesoporous silica nanoparticles	Type 1 diabetes	Insulin	~ 4 h	PBA-2 reacts with glucose, enabling selective insulin release	[25]
Dissolving microneedles	Hyaluronic acid	Poly(lactic-co-glycolic acid) nanoparticles,	Central nervous system diseases	Paroxetine, Rhodamine B	~ 12 h	HA dissolution releases the drug	[174]
Swelling microneedles	Hyaluronic acid	melanin nanoparticle	Skin tumor, Wound healing	SiO_4_^4−^	~ 15 days	Melanin NPs respond to NIR, generating SiO₄^4^⁻. ^−^	[35]
Swelling microneedles	Gelatin methacrylate, Polyethylene glycol diacrylate	MoS₂	Type 1 diabetes	Insulin	~ 3 h	MoS₂ responds to NIR, generating a photothermal effect to release insulin	[175]

**Fig. 13:**
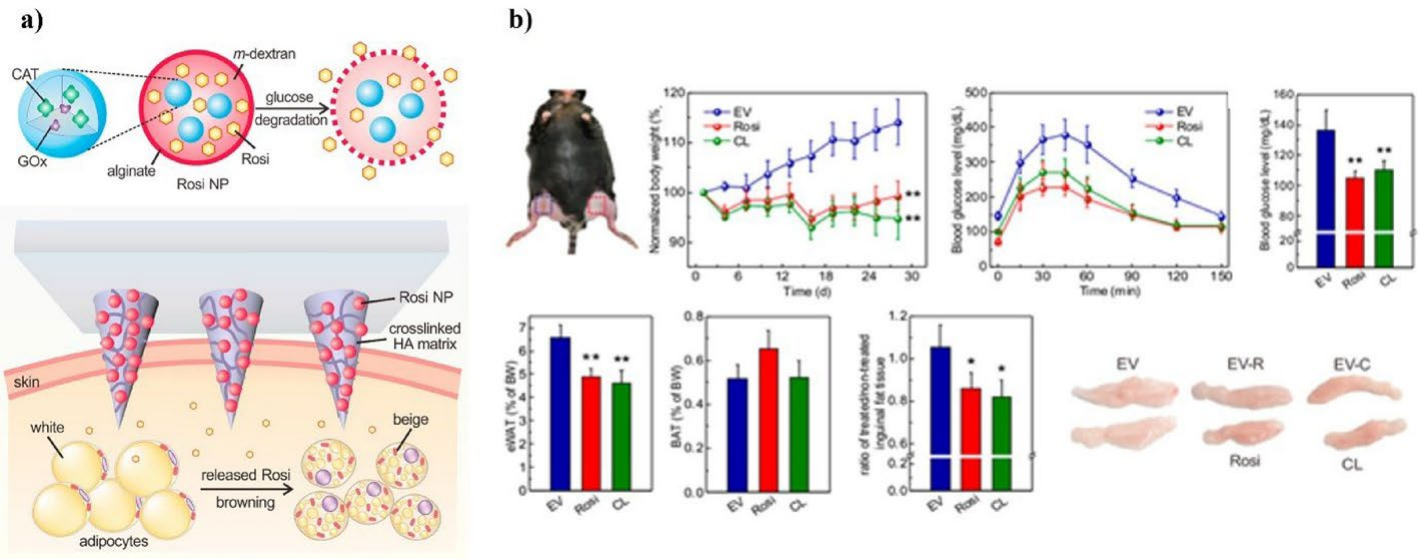
Obesity treatment via browning of white adipose tissue induced by dry powder and MnO₂ nanoparticles loaded in core–shell microneedles. **a** Scheme of percutaneous anti-inflammatory therapy using dry powder microneedles. **b** Relative weight changes, changes in the relative fat mass of subcutaneous inguinal WAT (IgWAT) and visceral epididymal WAT (EpiWAT), and fasting blood glucose concentration, relative lipid droplet area, and hepatic triglyceride content were assessed using high-fat diet (HFD)-induced obesity mouse models. Reproduced with permission from. Copyright (2024) Elsevier

Recent research has focused on treating obesity, a disease characterized by abnormal fat accumulation in the body. Obesity has been recognized as a public health problem, as it can cause diseases such as type 2 diabetes, cardiovascular disease, and cancer [36, 176]. Jun wu and Bruce M. Spiegelman et al. report that brown adipose tissue, a major thermogenic organ in mammalian energy expenditure, activates energy expenditure pathways using its ability to convert chemical energy into heat [177–180]. Based on these findings, research is underway to block energy accumulation as body fat, which is a major cause of obesity. A study by Yuqi Zhang et al. employed locally induced browning technology to convert white into brown adipose tissue as a potential treatment for obesity. In this study, the diabetes drugs Rosiglitazone and CL 316243 were loaded onto degradable nanoparticles and incorporated into microneedles (Fig. 13a) [20]. These finalized microneedle patches were tested in vivo on a lean mouse model, measuring caloric value, weight change, and oxygen consumption over four weeks. The results indicated that the patch reduced the growth of fat cells by about 15% after four weeks and improved how the body responds to insulin by lowering fasting blood sugar levels, showing its potential to boost metabolism (Fig. 13b). Ping Zhan and Peng Chen et al. also designed core–shell microneedles composed solely of compressed dry powder to achieve a loading capacity of 8 μg per microneedle, which is approximately twice the encapsulation capacity of conventional microneedles (10 ng per microneedle) [18]. The outer part of these core–shell microneedles is made of manganese dioxide nanoparticles (MnO₂-NPs), while the inside holds resveratrol (Res), a natural antioxidant that works well with MnO₂-NPs. To evaluate the therapeutic effect of the fabricated microneedles, a mouse model of obesity induced by a high-fat diet for two months was used. For 28 days, we administered Res-microneedles, MnO-microneedles, and MnO-Res-microneedles every other day to the mice. The results showed that while the control group of mice kept gaining weight, reaching 109.2% of their starting weight, the mice treated with Res-microneedles and MnO₂-microneedles had a noticeable drop in fat in both the fat under the skin and the fat around the intestines. Mice treated with MnO₂-Res microneedles weighed 14.2% less than the control group and demonstrated a significant decrease in fat mass. These results demonstrate that the microneedle patch minimized insertion stress and delivered a higher drug dosage compared to conventional nanoparticle technology while also providing enhanced drug efficiency and effective control over the drug release. These anti-obesity microneedle systems require thorough long-term assessments to confirm their safety and possible immunogenicity. Further adjustments are required to refine nanoparticle characteristics, such as size and surface charge, to achieve optimal tissue distribution while avoiding negative reactions. Incorporating real-time monitoring, like embedded biosensors, can enhance dosing regimens and boost treatment outcomes. Additional scaling studies and cost analyses are essential to assess the feasibility of manufacturing and distributing these patches on a larger scale. Novel microneedle–nanoparticle (MN–NP) devices have been proposed recently for the treatment of obesity. In their review of stimulus-responsive nanoparticle-integrated microneedle platforms intended to enhance site-specific delivery to adipose tissue, Chilamakuri et al. noted how these platforms could improve metabolic targeting and therapeutic retention, thereby overcoming the drawbacks of traditional anti-obesity therapies [181]. Furthermore, using controlled subcutaneous heat stimulation, Gao et al. created a photothermal microneedle patch with polydopamine nanoparticles that effectively caused white adipose tissue in obese mice to brown, resulting in a reduction of about 19% body weight [67].

**Fig. 14:**
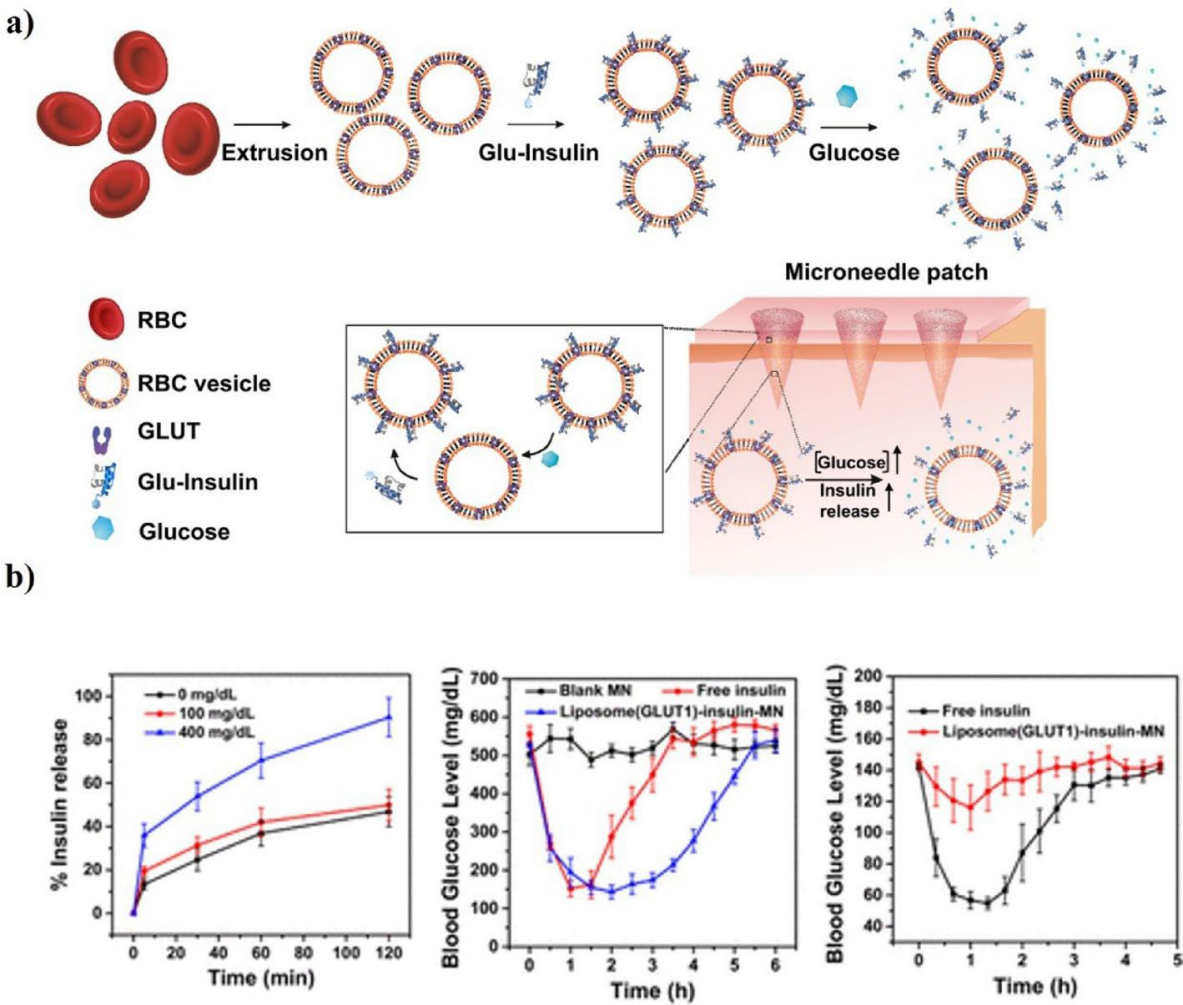
Regulated insulin delivery based on glucose-specific binding to Glu-Insulin. **a** Schematic of a glucose-responsive insulin delivery system using a microneedle patch conjugated with glucosamine-modified insulin (Glu-Insulin) and free Glu-Insulin in red blood cell (RBC) vesicles. **b** In vivo evaluation of Blank-MN, RBC-insulin-MN, and free insulin using an STZ-induced type 1 diabetes mouse model to determine the effectiveness of hyperglycemia treatment. Reproduced with permission from. Copyright (2022) ACS NANO

Among typical metabolic diseases, patients with hyperglycemia are steadily increasing because of poor eating habits. This rise is further exacerbated by lifestyle factors such as decreased physical activity and irregular meal patterns. Diabetes can be broadly categorized into two types: type 1 diabetes, which is characterized by the lack of insulin secretion from the pancreas, and type 2 diabetes, which involves increased insulin resistance despite insulin secretion from the pancreas, elevating the blood glucose levels [182–184]. In particular, patients with type 1 diabetes require insulin injections daily, which can be painful and provide a relatively short insulin duration. Recent studies have investigated using insulin-loaded nanoparticles within microneedles, which are expected to provide longer insulin release and reduce patient discomfort compared to conventional insulin injections. Yujie Zhang and Yifeng Lei, et al. developed single-use insulin microneedles. They utilized gold nanoclusters (AuNCs) to create a patch for treating type 1 diabetes, featuring glucose-responsive insulin release [185]. The microneedle patch is composed of biodegradable gelatin and starch. It is loaded with AuNCs that offer excellent drug-loading capacity and biocompatibility. The glucose-responsive AuNCs were synthesized using the CR9 peptide. These CR9-AuNCs have an insulin loading capacity of 1297 mmol per gram, which is exceptionally high. A therapeutic dissolving microneedle patch was fabricated to release a substantial quantity of insulin relative to its specific surface area in response to glucose. The microneedle patch was inserted into the dorsal skin of a type 1 diabetes-induced C57BL rat (eight weeks old) model to assess skin penetration, insulin release, and blood glucose-lowering effects. Results indicated that a single application maintained normal blood glucose levels in the type 1 diabetes rats for 1–2 days and alleviated typical symptoms of type 1 diabetes, such as increased hunger and thirst, frequent urination, and weight loss. Qian Chen and Zhen Gu, et al. fabricated a glucose transporter (GLUT) molecule-based patch containing GLUT1, the primary glucose receptor found in human red blood cells (RBCs), and GLUT4, the main glucose transporter in mouse RBCs[186]. The glucose-responsive microneedle patch for insulin delivery improved upon traditional insulin administration methods, which often did not align with physiological insulin secretion by β-cells (Fig. 14a). The RBC-insulin-microneedle patches were tested to see how well they could penetrate the skin and how effective they were in treating diabetes by measuring their strength, blood sugar levels, glucose tolerance, and insulin and glucose levels in male C57BL/6 type 1 diabetic mice aged 6 to 10 weeks. The results showed that the mechanical strength of the fabricated microneedles was 0.5 N/microneedle, compared to the minimum force required for skin penetration of 0.1 N/microneedle, indicating that the microneedles had sufficient mechanical properties. Blood glucose levels in mice treated with RBC-insulin-microneedles decreased rapidly within the first hour and remained approximately at 200 mg/dL without hypoglycemia for up to 5 h. IIn comparison, mice that received microneedles without insulin continued to have high blood sugar levels; their blood glucose was a bit lower than that of mice given free insulin alone, suggesting a lower chance of low blood sugar (Fig. 14b).Large-scale clinical validation remains essential, particularly regarding long-term immunogenicity and cost-effectiveness, to confirm these promising results. Incorporating closed-loop sensing components will enhance glucose-responsive insulin release by aligning it more closely with physiological needs. Enhanced mechanical strength and decreased tissue irritation from advanced composite materials may boost patient eligibility. Also, thorough studies on how often the patches can be used and monitoring them in real-time will reveal how effective these microneedle systems can be for managing diabetes every day. A pH-responsive microneedle system containing insulin nanoparticles was described by Lin et al. in relation to diabetes, and it produced glucose-sensitive, prolonged drug release in diabetic mice [136]. Furthermore, Liu et al. demonstrated accurate glycemic control in vivo by designing a closed-loop microneedle patch that combines glucose detection and insulin delivery by electroosmotic pumping [137].

**Fig. 15:**
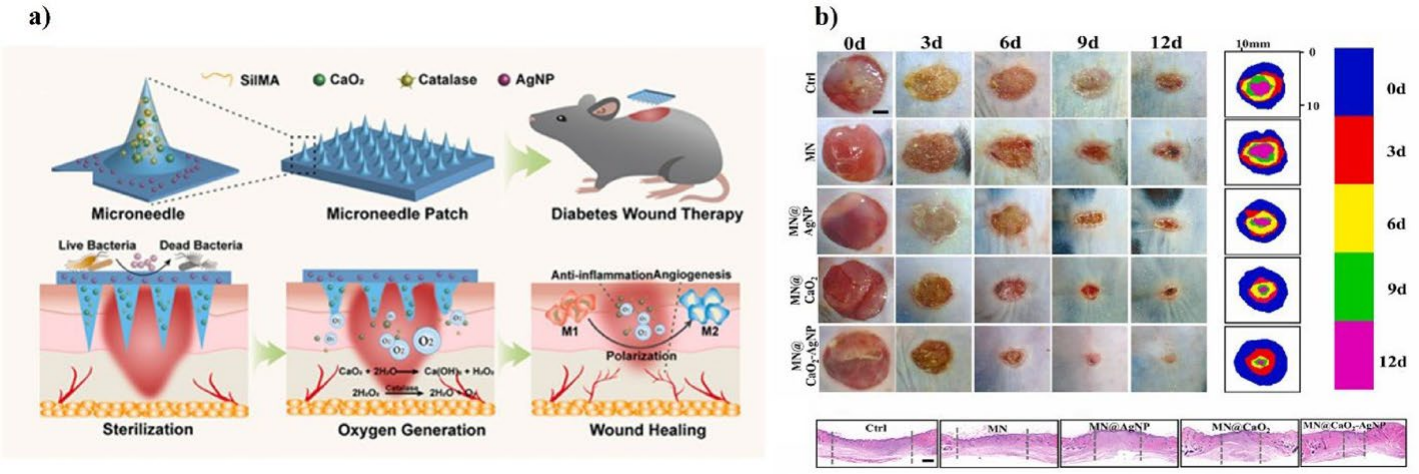
Fabrication of silver nanoparticle-encapsulated silk fibroin microneedle patches for the treatment of diabetic foot ulcers. **a** Schematic of the antimicrobial oxygen generation mechanism and microneedle patch design for diabetic wound healing. **b** Overall wound morphology and healing area throughout the treatment period across the five experimental groups. Hematoxylin & Eosin (H&E) staining results and changes in tissue thickness of the wound-healing tissues on day 12. Reproduced with permission from Copyright (2024) Elsevier

Wound healing occurs in four stages: hemostasis, inflammation, proliferation, and remodeling. However, in diabetic wounds, which are a diabetes complication, the natural wound healing rate is significantly slower compared with individuals with normal blood sugar. This delay is because of persistent inflammation, impaired angiogenesis, and increased oxidative stress [187–190]. Diabetic foot disease, which is particularly common in individuals with diabetes, is a complication where the skin or mucosal tissues suffer from inadequate blood supply and cannot heal naturally. This condition leaves them vulnerable to bacterial infections, which can ultimately require amputation of a part of the leg. Research on wound healing for these infections has revealed potential applications for chronic wound management and the treatment of diabetic foot ulcers. In a recent study, Xiaoling Lei et al. synthesized gelatin nanoparticles (GNPs) and conjugated AMP-Cypate to fabricate composite AMP-Cypate@GNPs [191]. The resulting microneedle patch exhibited high degradability and gelatinase-responsive release of the antimicrobial photothermal peptide AMP-Cypate. The microneedle patch was treated with a near-infrared laser (808 nm, 1.5 W/cm^2^) for 6 min on cultures of Staphylococcus aureus to test its antibacterial effectiveness, showing it could kill about 90% or more of the bacteria. To test how well it works in diabetic rats with long-lasting wounds caused by infections, oval wounds (8 × 6 mm) were made on the upper paws of the rats, infected with Staphylococcus aureus, and treated for about 20 days. The results showed that the PBS-treated group exhibited the highest scar formation compared with diabetic rats treated with PBS on day 20. On the other hand, the MN/AMP-Cypate@GNP + IR group displayed a significant amount of collagen fibers, suggesting a more robust healing response. CD31 staining was done to check for blood vessel growth, and it showed that the control group had much fewer blood vessels compared to both normal skin and the MN/AMP-Cypate@GNP + IR group. Therefore, the fabricated microneedles produced antibacterial patches with peptide release capabilities. Compared to conventional wound-healing patches, those made with microneedles and GNPs demonstrated superior wound-healing ability, showing a significant healing effect in the diabetic GK rat model. Adequate skin oxygenation is essential for rapid wound healing, as it supports cell growth and metabolism necessary for effective repair. A study by Mengli Sun et al. reported the development of a silk fibroin methacryloyl hydrogel microneedle patch, which incorporated a needle encapsulated with calcium peroxide and catalase and a base coated with antimicrobial silver nanoparticles (AgNPs) (Fig. 15a) [192]. TThe MN@CaO₂-AgNP patches were tested on eight-week-old C57BL/6 J mice to see how well they could stop bacteria from growing and invading diabetic wounds, help fix damaged cells and immune functions, and promote new blood vessel growth.The experimental groups included Control, MN, MN@CaO₂, MN@AgNP, and MN@CaO₂-AgNP. Results showed that wounds in the MN@CaO₂, MN@AgNP, and MN@CaO₂-AgNP groups healed effectively by day 3; the MN@CaO₂-AgNP group exhibited the most evident healing, as the wound area was significantly reduced by day 12. These findings suggest that calcium dioxide enhances skin healing, inhibits bacterial growth, and accelerates wound recovery in diabetic conditions (Fig. 15b). It remains uncertain whether extended exposure to nanoparticles may lead to local or systemic toxicity, considering the chronic nature of diabetic wounds. Additional studies are needed to assess the durability and mechanical stability of microneedle patches. Additionally, scalable manufacturing methods ensuring sterility and reliable performance will be essential for clinical translation. Future studies could integrate bioactive growth factors or oxygen-sensing features to enhance wound care for diabetic patients.

## Challenges and future directions

**Fig. 16:**
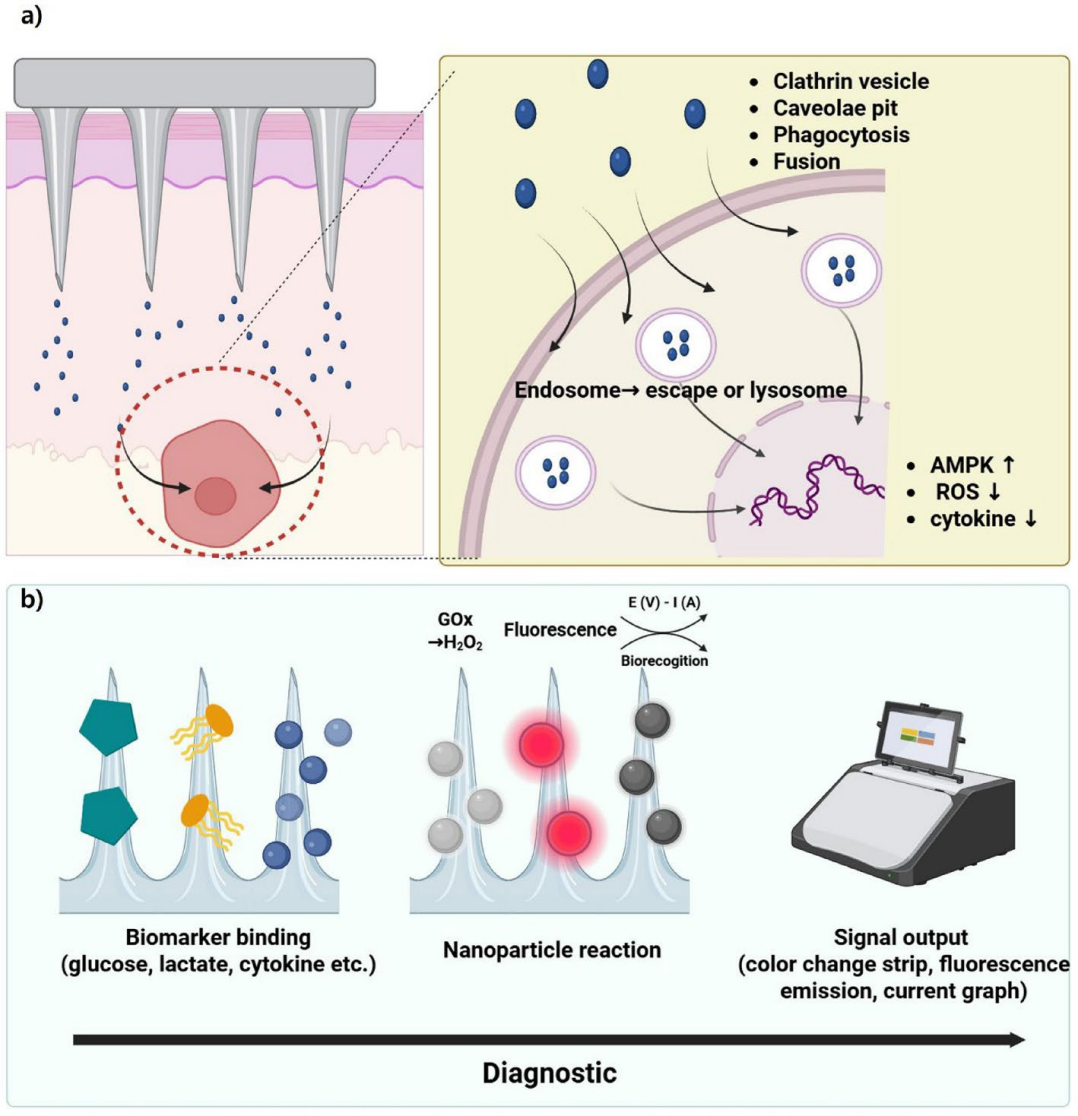
Schematic illustration of microneedle–nanoparticle mechanisms. **a** Nanoparticle delivery and cellular internalization pathways with downstream signaling. **b** Diagnostic workflow showing biomarker binding, nanoparticle reaction, and signal output (colorimetric, fluorescence, electrochemical)

We've already talked about how microneedle–nanoparticle devices have been used to deliver medicines and check for medical problems. This shows that they could improve treatments and make biomarker discovery possible in real time with little to no damage. In Fig. 16, you can see how microneedles and nanomaterials work together to control drug release and do sensitive biosensing for both healing and diagnostic purposes. Even though these changes show how adaptable these systems are, more work needs to be done to get past the problems that come up when they are translated and make sure they can be used safely in hospitals.

**Fig. 17:**
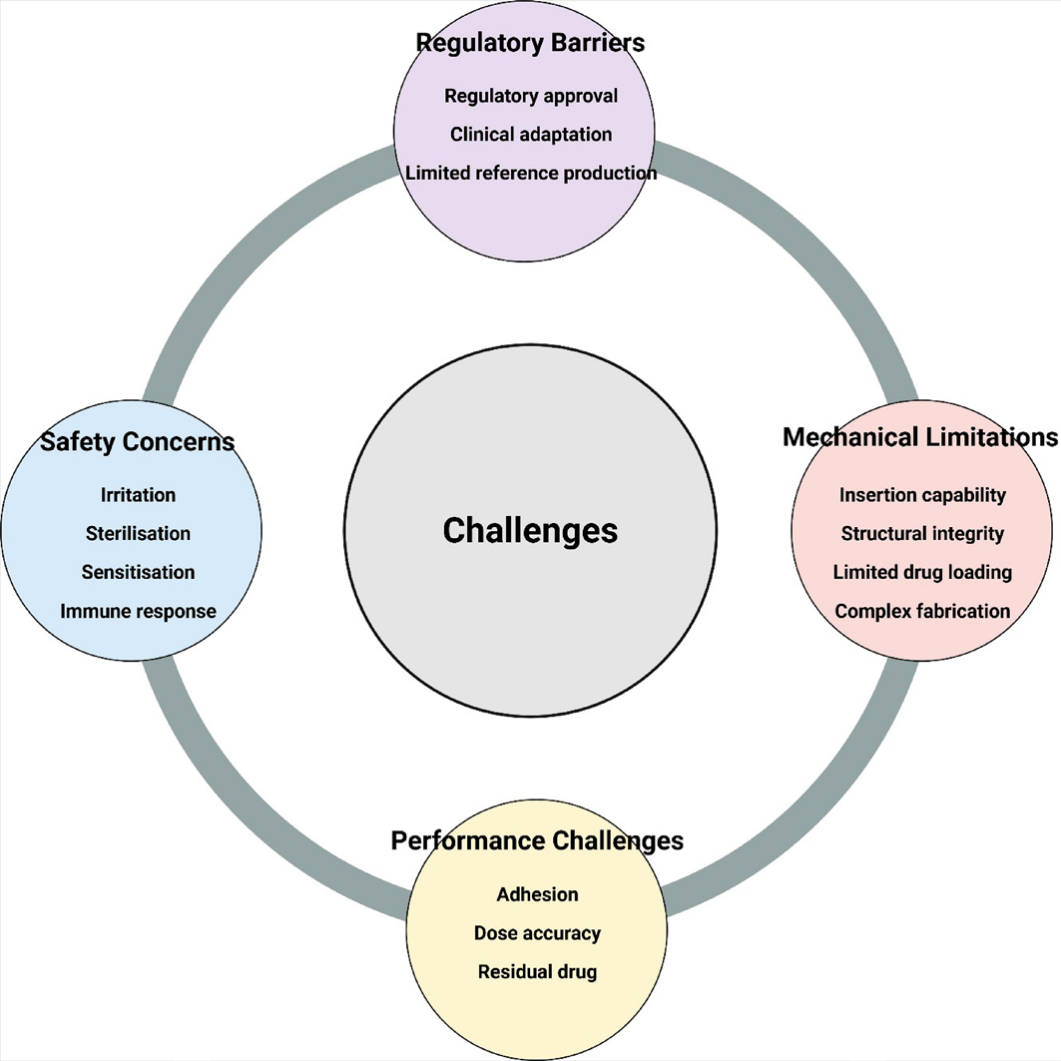
Significant obstacles to clinical adoption of microneedle–nanoparticle systems. Safety, mechanical strength, performance dependability, and regulatory complexity are important considerations. Created with BioRender.com

Despite recent progress in microneedle-nanoparticle integrated systems, considerable obstacles persist, especially concerning biocompatibility, toxicity, and clinical use. Recent studies highlight possible issues related to the long-term safety of nanoparticles and microneedle materials, underscoring the need for thorough evaluations of cytotoxicity, genotoxicity, and immunogenicity by extensive in vitro and in vivo investigations. Recent clinical studies have identified particular translational challenges, such as negative immunological responses and systemic toxicity arising from nanoparticle breakdown products, requiring thorough assessment of their metabolic pathways and excretion profiles to guarantee patient safety. The complexity of regulatory approval processes for combination devices, including the demonstration of repeatability, efficacy, and long-term stability, remains a substantial obstacle to prompt clinical use. Clinical trials utilizing microneedle-nanoparticle systems encounter additional issues stemming from rigorous patient inclusion criteria, difficulties in patient recruiting, and extended follow-up durations, which collectively impede the translational process. Patient adherence is a vital factor in clinical success; new research highlights that device acceptability significantly depends on user-friendly design, low discomfort, ease of use, and evident therapeutic results. Addressing these complex challenges necessitates interdisciplinary collaboration among researchers, healthcare practitioners, regulatory bodies, and industry stakeholders, promoting a cohesive strategy to expedite the clinical application of microneedle-nanoparticle therapeutic platforms. Figure 17 graphically highlights these translational challenges, emphasizing device adherence, dosage precision, mechanical integrity, payload capacity, immunogenicity, cytotoxicity issues, and regulatory approval complications.

The efficacy of signal transduction, together with specificity and sensitivity, remains a considerable problem in microneedle–nanoparticle hybrid diagnostic devices. Efficient signal transduction necessitates precise interactions between nanoparticles and target biomarkers, converting biochemical interactions into measurable electrical, optical, or colorimetric signals. Achieving high specificity is particularly challenging due to potential cross-reactivity with structurally similar molecules or interfering substances in interstitial fluid, necessitating careful optimization of biorecognition elements such as antibodies, aptamers, or enzymes. Sensitivity, defined as the minimal detectable concentration of the biomarker, significantly influences clinical application. Enhancing sensitivity often requires meticulous adjustment of nanoparticle attributes, such as particle size, surface charge, and functionalization density, which directly affect biomarker binding affinity and signal amplification capacity. Recent studies have utilized plasmonic nanoparticles and enzyme-mimetic nanozymes to enhance sensitivity, demonstrating the potential to reach detection limits in the picomolar to femtomolar range [94, 95]. Despite these advancements, maintaining signal stability throughout ongoing monitoring and reducing noise and background interference continue to be enduring issues. Future research efforts should focus on the advancement of complex nanomaterial interfaces, the implementation of novel surface chemistries, and the application of machine learning algorithms for accurate signal interpretation to resolve these challenges and facilitate successful clinical translation.

### Nanomaterial design considerations and limitations

Adding nanomaterials to microneedle platforms could make treatments work much better and make diagnosis much more accurate. But there are still a lot of flaws that need to be fixed before they can be utilized in hospitals. Biocompatibility and the management of degradation products provide significant challenges. PLGA and other biodegradable polymers decompose into monomers that the body may utilize. In contrast, inorganic carriers and cationic polymers may generate harmful by-products, raising apprehensions regarding long-term accumulation and toxicity [193]. These dangers show how important it is to do more thorough toxicological tests, especially when the same drug is given more than once. Immunogenicity makes things more difficult since microneedles with nanoparticles in them may cause unwanted immune responses over time. Regulatory procedures are highly tight, so you need to be able to reproduce the results, make a lot of copies, and have a lot of long-term safety data to get permission. The absence of established criteria for microneedle–nanoparticle hybrids impedes their application in therapeutic contexts [194]. The way something functions and how safe it is also dependent on its physicochemical properties. Controlling the size and surface charge of particles impacts not just how they get through the skin and disseminate through interstitial fluid, but also how they get into the body and how they are eliminated. Smaller nanoparticles get through better, but they could get into the bloodstream and build up in organs that aren't the target. Larger or positively charged systems, on the other hand, improve local retention but could stick to other things and be bad for cells [195]. The shape of the particles and the chemistry of their surfaces give designers more choices, but they also make it difficult to guess what will happen in vivo. PEGylation, for instance, makes medications stay in the body longer, but it can also make the body generate anti-PEG antibodies after a few doses. Biomimetic coatings are promising, but they can be hard to forecast because of where the biological membranes come from. These trade-offs highlight how hard it is to make nanomaterials that are both safe and useful. The reasons for combining certain nanomaterials with certain microneedle designs are just as essential. Polymeric nanoparticles that break down with the microneedle matrix help break down microneedles. Strong metallic or inorganic carriers work better with solid and hollow microneedles, on the other hand. Microneedles that make hydrogels create new ways to continuously sense things and control the release of enzymes or compounds that can conduct electricity. But it's impossible to anticipate how much they will swell. Stimuli-responsive microneedles demonstrate the capabilities of advanced nanomaterials; yet, their dependence on highly specialized chemistries presents challenges for scalability and regulatory approval [70].So, choosing a material can't only be based on how easy it is to get or how easy it is to make; it needs to be based on a deep understanding of how nanoparticles behave, how microneedles work, and what the therapeutic needs are. All of these problems show that designing nanomaterials for microneedle systems is not only a technological challenge but also a regulatory, biological, and translational problem. We need to manufacture new materials, make testing methods the same, perform long-term safety research, and make the rules clearer to fix these problems. Microneedle platforms that use nanomaterials will never be able to do what they can do in the clinic if these things don't happen.

### Immunological responses, skin irritation, and biocompatibility

**Fig. 18:**
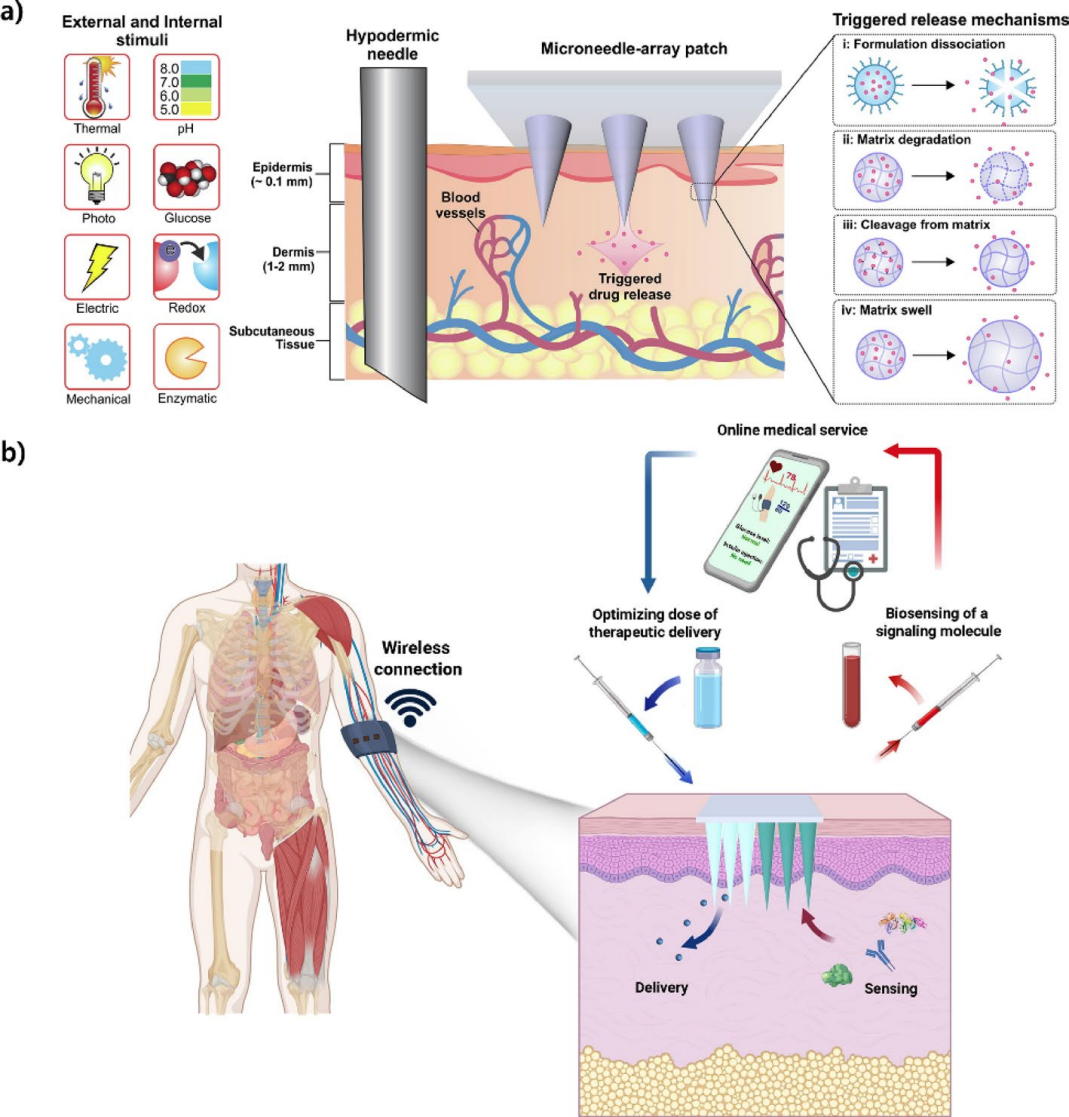
Stimuli-responsive microneedle platforms for intelligent transdermal therapy. **a** Overview of internal and external stimuli (e.g., pH, thermal, photo, enzymatic) used to trigger drug release from microneedle-array patches via mechanisms such as formulation dissociation, matrix degradation, and swelling. **b** Concept of closed-loop theranostic microneedles integrating biosensing and controlled delivery, wirelessly connected to online medical services for real-time diagnosis and dose optimization. Adapted with permission from Zhao et al., Copyright (2021), Materials Today

Recent advances in microneedle technology, including stimuli-responsive devices and biosensing wearable patches, have significantly enhanced individualized treatment for metabolic disorders. Stimuli-responsive microneedles utilize internal biological indicators, such as glucose concentrations, pH variations, and enzyme activity, in conjunction with external physical stimuli, including photonic, thermal, and electrical signals, to provide precise and personalized drug delivery. Recent studies indicate that incorporating nanomaterials such as glucose-responsive polymeric nanoparticles or gold nanoparticles into advanced formulation techniques markedly improves therapeutic efficacy and target specificity compared to traditional microneedles alone. Wearable diagnostic microneedle systems using biosensing nanoparticles markedly enhance sensitivity, response times, and continuous real-time metabolic monitoring. These integrated devices facilitate wireless data transmission to healthcare professionals, allowing for real-time dosage adjustments according to each patient's metabolic profile (Fig. 18). Despite these promising technological advancements, questions remain regarding device accuracy, reliability, possible immune reactions, and skin irritation. A recent study underscores the imperative of addressing these substantial challenges through standardized safety assessments, rigorous clinical validation, and adherence to regulatory standards to ensure patient safety and treatment efficacy. Future research must focus on mitigating these limitations, particularly by enhancing material properties, optimizing biocompatibility assessments, and conducting controlled clinical trials [196].

### Future perspectives

Through minimally invasive and programmable interventions, microneedles and nanoparticles hold great promise for revolutionizing the detection and treatment of metabolic disorders [197]. At the same time, new biomaterials are being developed to address the issues of toxicity, immunogenicity, and skin irritation brought on by frequent use of MN. Examples of these include stimuli-responsive hydrogels, zwitterionic polymers, and biodegradable nanocomposites [198]. New material platforms are making it possible to design microneedle–nanoparticle systems in new ways. Metal–organic frameworks (MOFs), such as ZIF-8 and bimetallic/biometal variants, provide very high loading and catalytic/ROS-modulating functions for antibacterial therapy and stimuli-responsive delivery [199, 200]. They have even made it possible to make wearable Eu-MOF microneedle patches for fluorescence sensing, which shows how useful they could be for diagnosis. COFs, which are crystalline porous organic networks, offer light-element frameworks with strong photothermal and chemotherapeutic co-delivery. A COF-based microneedle patch has recently shown to be effective against tumors in vivo [201, 202]. Conductive polymers (e.g., PEDOT:PSS) incorporated with hydrogel microneedles provide low-impedance interfaces and enzyme-free, real-time electrochemical detection of metabolites such as glucose, indicating stable, on-skin signal transduction for continuous monitoring [203]. These materials collectively indicate prospects for multiplexed diagnostics and combination therapy; however, challenges such as long-term stability, potential metal-ion leaching in metal–organic frameworks (MOFs), batch-to-batch variability in covalent organic frameworks (COFs), and the scalability and biocompatibility of conductive composites persist as significant obstacles to clinical translation.

Recently, cost-effective, high-fidelity MN-NP systems with enhanced repeatability and quick prototyping have been made possible by scalable microfabrication techniques such as 3D printing, soft lithography, and laser micromachining [204]. In the end, clinical translation will necessitate the creation of strong regulatory frameworks, usability testing in a range of patient demographics (such as children and the elderly), and integration with cloud data systems and telemedicine [205]. Interdisciplinary partnerships spanning nanotechnology, material science, data analytics, and clinical care will be crucial for implementing patient-centered MN-NP systems for future metabolic healthcare deployment.

**Fig. 19:**
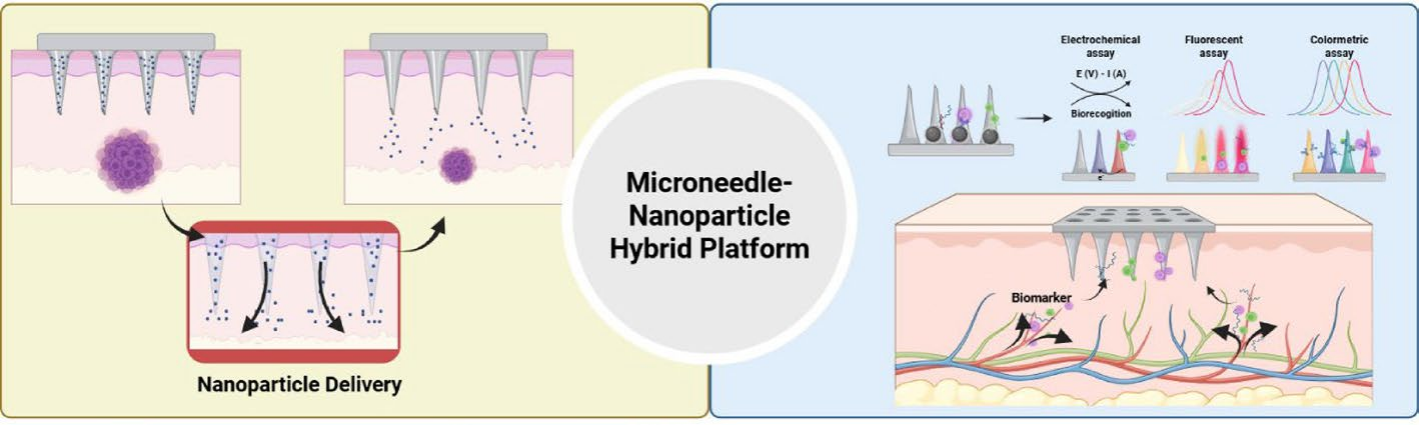
A detailed mechanistic diagram elucidating the operational workflow and interaction mechanisms of the microneedle–nanoparticle hybrid system. (1) the penetration of microneedles into the skin and formation of microchannels; (2) release or extraction of nanoparticles via these channels; (3) nanoparticle interaction with target biomarkers in interstitial fluid (for diagnostics) or controlled therapeutic payload release (for therapeutics); and (4) the signaling mechanisms for diagnostic readout (optical, electrochemical, or colorimetric)

To visually illustrate the integrated operation of microneedle–nanoparticle systems, a schematic representation of their working principles is presented (Fig. 19). This includes both the controlled release of therapeutic nanoparticles through microneedle-induced microchannels and the capture and detection of biomarkers using nanoparticle-functionalized microneedles in combination with electrochemical, fluorescent, or colorimetric assays. Such dual-functional platforms highlight the potential of microneedle–nanoparticle hybrids as versatile tools for simultaneous diagnosis and treatment in point-of-care settings.

## Conclusion

This review briefly summarizes the recent research on integrating nanoparticles and microneedles for diagnosing and treating metabolic syndrome. This fusion strategy can overcome the disadvantages of conventional transdermal administration, such as non-invasiveness, limited drug delivery, and controlled drug release [206]. It can also reduce drug-induced systemic toxicity and increase therapeutic efficacy, thereby expanding nanomedicine applications [207]. In addition to being an effective and safe drug delivery system, microneedles fused with these nanoparticles have been widely studied for diagnosing and treating cancer and skin diseases because of their simplicity and sensitivity in the diagnostic field.

Despite this promising potential, the fusion of nanoparticles and microneedles still presents challenges that need to be addressed [208]. These include ensuring the long-term safety and biocompatibility of materials used and addressing technical complexities associated with the precise fabrication and consistent performance of these devices. The materials used in both microneedles and nanoparticles must be guaranteed to be biocompatible. Prolonged exposure to these substances should not cause adverse immune reactions or toxicity. Research should focus on comprehensive biocompatibility testing, including in vitro and in vivo studies, to assess the potential cytotoxicity, genotoxicity, and immunogenicity. Degradation products of both microneedles and nanoparticles should be nontoxic. For example, dissolving microneedles should dissolve completely without leaving harmful residues; nanoparticles should be designed to degrade into nontoxic byproducts that can be safely metabolized or excreted from the body. Precise manufacturing with consistent dimensions, clarity, and mechanical strength is critical for microneedle performance. Incorporating nanoparticles into these structures adds another layer of precision. Technologies such as micromachining and three-dimensional printing must be developed and optimized to achieve high precision and repeatability. Nanoparticle stability within the microneedles must be maintained to ensure that they do not aggregate or disintegrate over time, which can affect nanoparticle efficacy and safety. Advanced formulation strategies are required to maintain the integrity of these nanoparticles and their fusion with microneedles. Efficient and controlled therapeutic delivery from nanoparticles to the target tissue via microneedles is essential. This requires fine-tuning the release kinetics and ensuring that the therapeutic payload remains active and bioavailable upon delivery. Overall, the convergence of microneedle and nanoparticle technologies offers an effective approach to managing metabolic diseases with the potential to completely changes current treatment paradigms. As research in this field continues to advance, relevant issues must be thoroughly investigated and addressed to develop safe, effective, and patient-centered therapeutic strategies.

## Supplementary Information

Additional file 1 (DOCX 723 KB)

## Data Availability

No datasets were generated or analysed during the current study.

## Abbreviations

ACG: Acetylated cashew gum
CNC: Cellulose nanocrystal
CNT: Carbon nanotube
EMNs: Emerging microelectronic microneedles
GNPs: Gelatin nanoparticles
Gox: Glucose oxidase
GSH: Glutathione
H₂O₂: Hydrogen peroxide
HA: Hyaluronic acid
HRP: Horseradish peroxidase
ISF: Interstitial fluid
MCM: Mixed valence cerium-metal organic frame
MTX: Methotrexate
PANI: PH-responsive polyaniline
PDMS: Polydimethylsiloxane
PLA: Polylactic acid
TMB: 3,3',5,5'-Tetramethylbenzidine
