# Supplementary figures and images for: Microneedle–nanoparticle hybrid platforms for metabolic syndrome: advances in point-of-care diagnostics and transdermal therapeutics
Source: Discov Nano. 2025 Oct 28;20(1):193. doi: 10.1186/s11671-025-04376-7 (PMC12569312; doi:10.1186/s11671-025-04376-7)

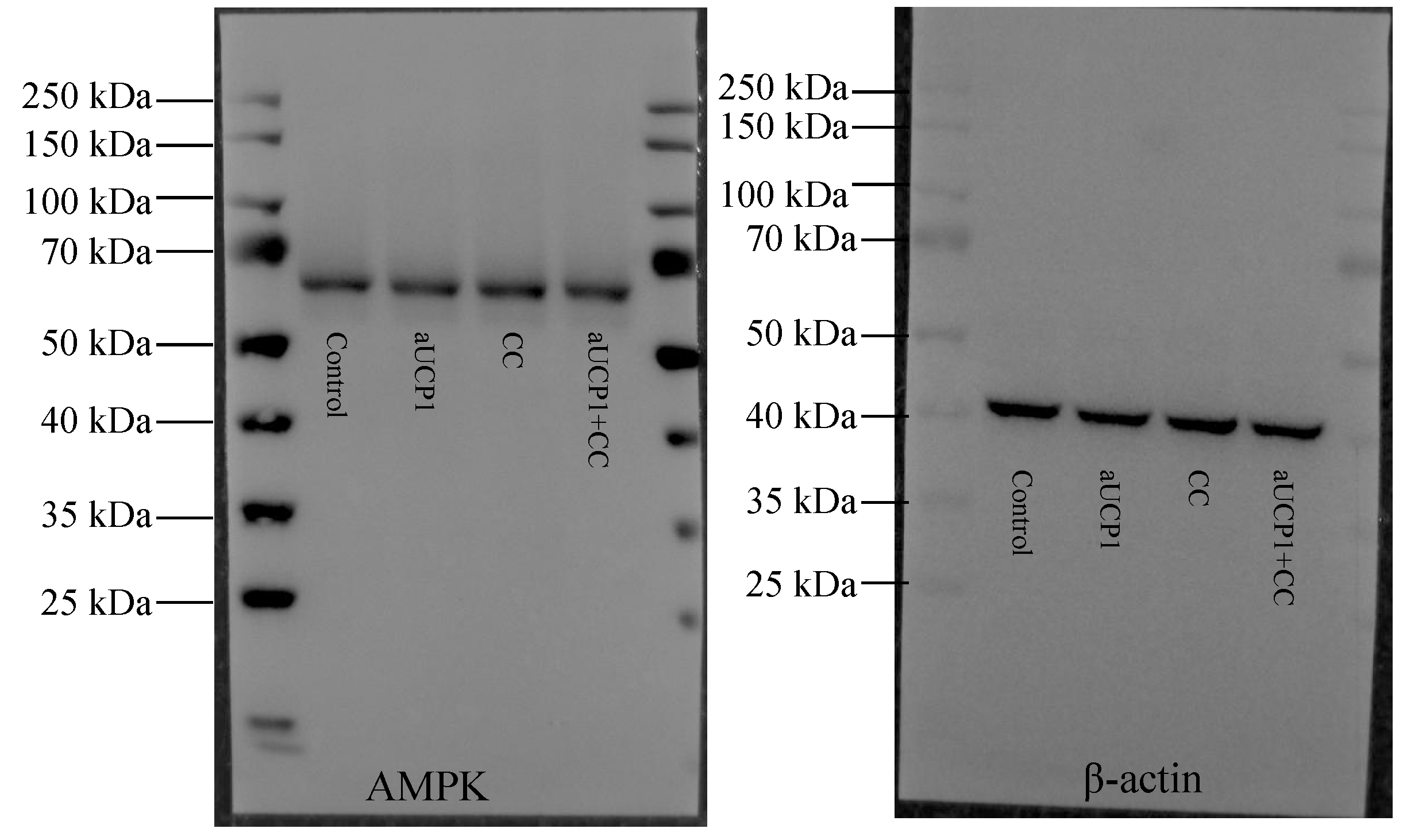

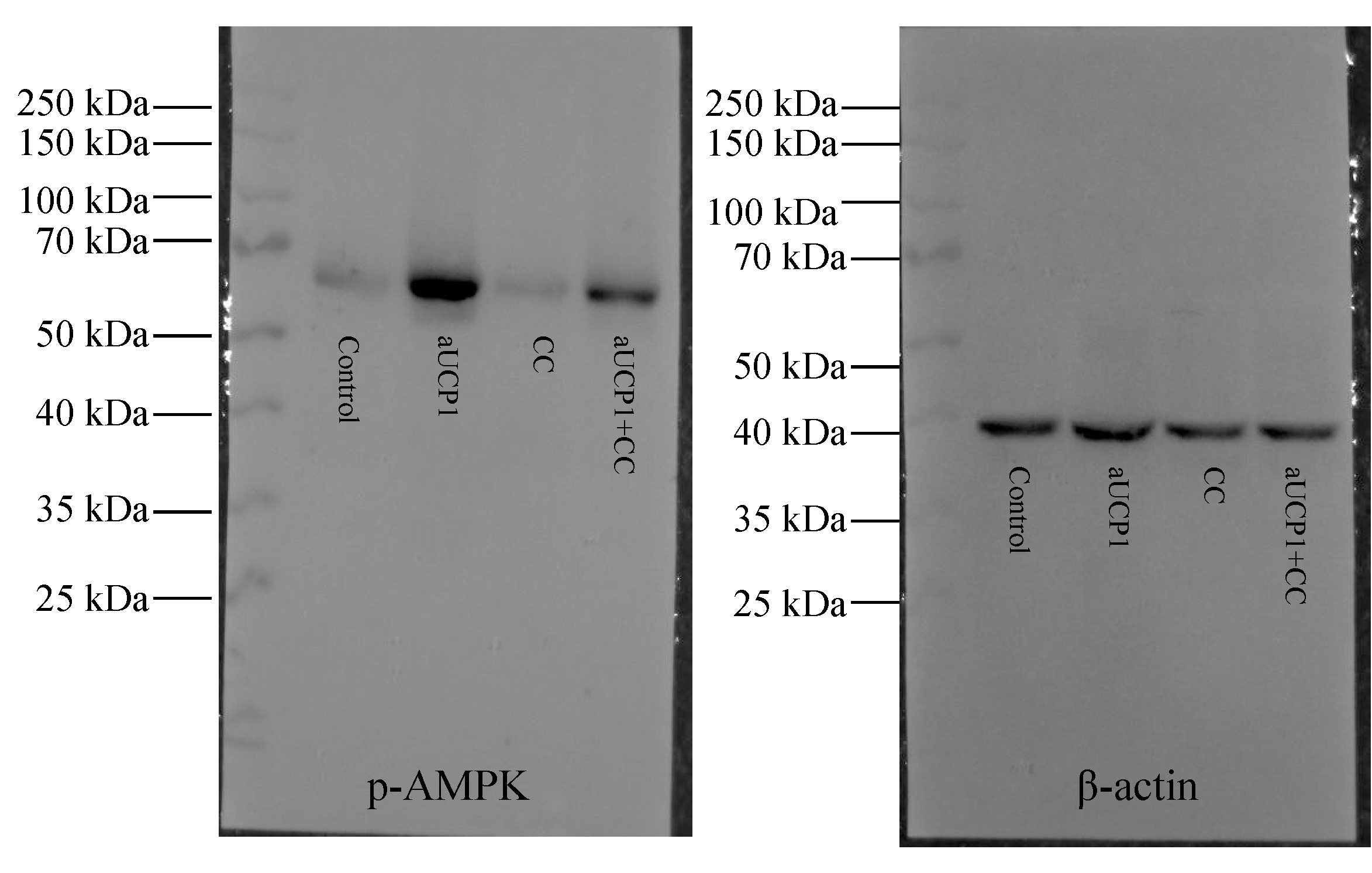

Supplement: Supplementary file 1 — Additional file 1 (DOCX 723 KB) [file 11671_2025_4376_MOESM1_ESM.docx]
